# Triple-emulsion microfluidic Core–Shell hydrogel microcapsules for oral pentoxifylline Delivery: Ameliorating colitis and rebalancing gut microbiome

**DOI:** 10.1016/j.mtbio.2026.102881

**Published:** 2026-02-02

**Authors:** Ji-Yeon Park, Hye-Seon Jeong, Seong-Ryeong Lim, Won-Kyo Jung, Jae-Young Je, Chang-Hyung Choi, Sei-Jung Lee

**Affiliations:** a Major of Human Bio-convergence, Division of Smart Healthcare, Pukyong National University, Busan, 48513, Republic of Korea; b School of Chemical Engineering, Yeungnam University, 280 Daehak-ro, Gyeongsan, Gyeongbuk, 38541, Republic of Korea; c Major of Biomedical Engineering, Division of Smart Healthcare, Pukyong National University, Busan, 48513, Republic of Korea

**Keywords:** PTX, Oral delivery, PHM, Gut microbiota, IBD

## Abstract

**Inflammatory bowel disease (IBD) encompasses chronic or relapsing inflammation within different regions of the gastrointestinal tract. Pentoxifylline (PTX)**, a methylxanthine derivative primarily used to improve blood flow in peripheral vascular diseases, has demonstrated anti-inflammatory and immunomodulatory properties, suggesting its potential in attenuating IBD-associated inflammation. However, its clinical application in IBD remains limited, partly due to its short half-life and poor targeting to inflamed intestinal tissues, necessitating strategies to enhance its bioavailability and tissue-specific delivery. To address this limitation, we developed a targeted drug delivery system utilizing a microfluidic approach to fabricate pH-responsive core–shell hydrogel microcapsules encapsulating PTX, referred to as PTX-loaded hydrogel microcapsules (PHM), for enhanced delivery to inflamed colonic tissue. These microcapsules were generated via photopolymerization of triple emulsion droplets, resulting in a structure composed of a poly (acrylic acid)-poly (ethylene glycol) diacrylate (PAA–PEGDA) shell and a PEGDA core, separated by a thin oil layer. The oil layer serves as a protective barrier against the acidic gastric environment, while the pH-responsive swelling of the PAA–PEGDA shell at basic pH (7.5) compresses and destabilizes the oil layer, thereby enabling controlled PTX release specifically in the colonic environment. *In vivo* studies using dextran sulfate sodium (DSS)-induced IBD in ICR mice demonstrate that PHM significantly mitigates disease severity, as evidenced by an approximately 38.5% reduction in disease activity index scores, restoration of mucosal architecture, and decreased infiltration of colonic macrophages. In parallel, PHM treatment markedly suppresses colonic inflammatory responses, lowering IL-1β by 40%, IL-6 by 66.2%, and TNF-α by 36.2% compared to DSS-treated mice, along with a broader reduction of pro-inflammatory mediators, highlighting its anti-inflammatory potential. Notably, PHM also contributes to the rebalancing of dysbiotic gut microbiota, including the restoration of beneficial genera such as *Bacteroides acidifaciens* and *PAC001120_s*, thereby promoting microbial homeostasis. Collectively, these findings underscore PHM as a promising PTX-based therapeutic strategy for effective IBD intervention.

## Highlights

• PTX-loaded pH-responsive core–shell microcapsules (PHM) with an oil layer are fabricated via triple-emulsion microfluidics.
• Oral PHM enhances PTX stability and enables colonic delivery under intestinal pH conditions.
• Released PTX alleviates intestinal inflammation in a DSS-induced colitis model.
• PHM mitigates gut dysbiosis by reducing *B. acidifaciens* and enriching SCFA-producing taxa.

## Graphical abstract

Graphical Abstract. Pentoxifylline delivery via pH-responsive core–shell microcapsules alleviates inflammatory bowel disease and restores gut microbiome dysbiosis. A pH-responsive core–shell hydrogel microcapsule system (PHM) is engineered via a microfluidic triple emulsion process for the oral delivery of pentoxifylline (PTX) to inflamed colonic tissue in an IBD mouse model. The microcapsule consists of a porous PAA-PEGDA shell, an internal PEGDA core, and a protective oil barrier that remains stable in the acidic gastric environment (pH 2). Upon exposure to colonic pH (pH 7.5), the PAA shell transitions into a stretched form, compressing the capsule structure and triggering oil barrier dewetting to enable controlled PTX release. Following oral administration in an IBD model, PHM prolongs gastrointestinal transit and enhances colonic delivery of PTX, supporting its controlled release and mucosal retention in the inflamed intestine. This spatiotemporal distribution correlates with intestinal kinetics and contributes to decreased macrophage infiltration, reduced expression of inflammatory mediators, and recovery of epithelial architecture. Simultaneously, PHM modulates the gut microbiota by suppressing *Bacteroides acidifaciens*, an inflammation-associated mucin-degrading species, and enriching *Lachnospiraceae*-related PAC001120, a beneficial SCFA-producing taxon. Collectively, these effects contribute to attenuation of intestinal inflammation and microbial dysbiosis, thereby alleviating pathological features of IBD.

**Figure:**
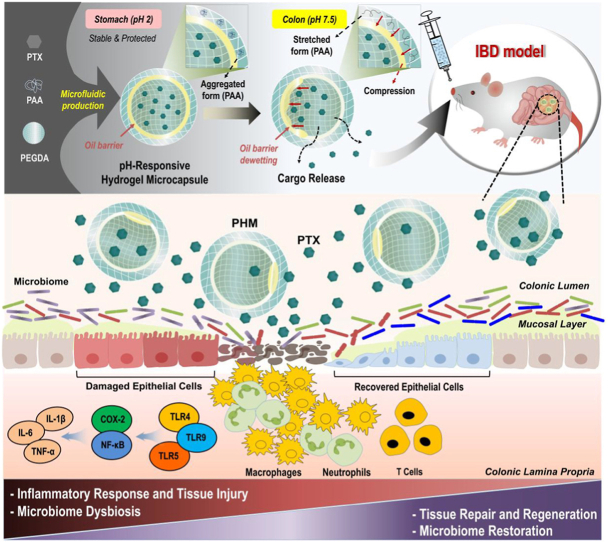

## 1 Introduction

Disruptions in the intestinal immune response and alterations in the gut microbiota composition play a central role in the initiation and progression of inflammatory bowel disease (IBD) [1]. IBD is marked by chronic or relapsing inflammation along different segments of the gastrointestinal tract, often manifesting as persistent diarrhea, rectal bleeding, abdominal discomfort, and unintended weight loss [1]. Although the precise etiology of IBD remains unclear, current evidence points to a dysregulated immune response in genetically predisposed individuals, triggered by host-associated microbiota and exacerbated by compromised epithelial and mucosal immune barrier functions [1,2]. Conventional pharmacological treatments—including aminosalicylates, corticosteroids, and immunomodulators—frequently produce adverse effects and demonstrate limited efficacy, largely due to their non-specific anti-inflammatory mechanisms [3]. This underscores an urgent need for more targeted and effective therapeutic strategies in the clinical management of IBD. Interestingly, recent findings suggest that IBD shares key mechanistic features with other chronic inflammatory disorders such as atherosclerosis, chronic venous insufficiency, type 2 diabetes, and non-alcoholic fatty liver disease [[4], [5], [6]]. These diseases commonly involve persistent immune dysregulation, endothelial dysfunction, and systemic low-grade inflammation. In addition, growing evidence implicates disruptions in the gut–vascular axis and aberrant cytokine signaling as contributors to the exacerbation of intestinal inflammation and tissue damage in IBD [7,8]. These shared pathways present a compelling rationale for exploring therapeutic interventions that concurrently modulate both systemic and mucosal inflammation, thereby offering a novel and more holistic approach to IBD treatment.

Pentoxifylline (PTX), a methylxanthine derivative initially used to improve peripheral circulation in conditions like intermittent claudication, has recently emerged as a potential therapeutic agent for IBD [[9], [10], [11]]. In addition to its vascular benefits, it exhibits anti-inflammatory, immunomodulatory, and anti-fibrotic effects, which may be beneficial in IBD management [10,12]. PTX reduces intestinal inflammation by inhibiting TNF-α, suppressing NF-κB activation, and lowering levels of pro-inflammatory cytokines such as IL-1β and IL-6—all implicated in IBD pathogenesis [12,13]. It also enhances microcirculation and reduces oxidative stress, promoting mucosal healing and epithelial integrity [11]. Moreover, preclinical studies have shown that PTX can **modulate immune cell infiltration**, attenuate tissue fibrosis, and ameliorate histological damage in models of chronic colitis, further supporting its therapeutic potential [10,11]. Compared to standard IBD therapies, PTX shows a more targeted anti-cytokine profile and is generally well tolerated, with side effects like nausea or dizziness being mild and dose-dependent [14]. Despite these advantages, the oral administration of PTX presents pharmacokinetic and safety-related challenges in the context of IBD. Its relatively short half-life, rapid systemic clearance, and limited retention at inflamed intestinal sites often necessitate higher or more frequent dosing to achieve sustained therapeutic effects [14]. However, dose escalation can increase systemic exposure and broaden off-target distribution, potentially heightening the likelihood of dose-dependent adverse events and compromising long-term tolerability [15]. In clinical use, PTX-related side effects are most commonly gastrointestinal (e.g., nausea, dyspepsia), but systemic effects such as dizziness, headache, and cardiovascular symptoms (e.g., flushing or hypotension) have also been reported, particularly at higher doses or in sensitive individuals [16,17]. Moreover, because IBD is a chronic, relapsing–remitting disease that may require repeated treatment cycles, cumulative systemic exposure and off-target effects become important considerations for patient adherence and overall safety. Therefore, targeted oral drug delivery strategies that enhance local PTX bioavailability at inflamed mucosal sites—while limiting premature release in the upper gastrointestinal tract and minimizing systemic absorption—are urgently needed to unlock the full therapeutic potential of PTX in IBD.

The gastrointestinal microenvironment in IBD exhibits pronounced spatial and temporal heterogeneity in pH rather than a uniform acidic condition [18]. Acute inflammatory lesions are often accompanied by localized acidification driven by immune cell infiltration, hypoxia, and metabolic reprogramming, whereas the intestinal lumen—particularly in the ileum and colon—undergoes dynamic pH fluctuations during disease progression and resolution [18]. Recent studies have further linked this luminal pH variability to inflammatory activity, epithelial dysfunction and repair, and alterations in microbial composition, resulting in distinct local microenvironments across different disease stages [19,20]. Importantly, as inflammation subsides or therapeutic intervention restores epithelial and microbial homeostasis, luminal pH tends to shift toward near-neutral or slightly alkaline conditions [18,20]. Given the relapsing–remitting nature of IBD, such pH transitions recur over cycles of disease exacerbation and remission, creating a dynamic physiological window that can be exploited for rational drug delivery design. From a therapeutic perspective, this pH heterogeneity underscores the need for oral delivery systems that remain stable under harsh gastric and inflammatory conditions while enabling controlled release under the milder pH environments of the distal intestine or recovering colon. Accordingly, recent biomaterials research has emphasized delivery platforms that balance protection during gastrointestinal transit with pH-responsive activation at neutral or slightly alkaline conditions relevant to colonic disease sites [21,22]. Biomaterials designed to resist acidic degradation while permitting site-specific release offer clear advantages for IBD therapy, although achieving both robust protection and precise stimulus-responsive release remains a challenge. Thus, rational design of pH-responsive biomaterials that account for the spatiotemporal pH landscape of IBD is increasingly recognized as critical for effective oral therapeutics.

To overcome these limitations, advanced encapsulation strategies have been developed to shield therapeutic agents from the harsh gastrointestinal environment, thereby improving their chemical stability, mucosal penetration, and systemic bioavailability. These innovations enable lower dosing regimens while preserving therapeutic efficacy, ultimately reducing the risk of dose-dependent side effects. Among these technologies, droplet-based microfluidics has emerged as a powerful tool for generating highly uniform multiphase emulsions, which serve as templates for constructing advanced drug carriers such as liposomes [23], polymersomes [[24], [25], [26]], and core-shell microcapsules [27,28]. These systems have been designed to optimize drug delivery efficiency while minimizing adverse effects. While microfluidically produced liposomes and polymersomes are effective at encapsulating hydrophilic compounds [29], their low mechanical stability under physiological conditions can result in membrane rupture or collapse, limiting their long-term functionality *in vivo*. Consequently, research has shifted toward the use of multi-compartmental microcapsules, which offer enhanced structural integrity and customizable architectures for encapsulating diverse therapeutic agents [30,31]. In particular, double emulsions have proven useful as templates for creating microcapsules capable of controlled and simultaneous release of both hydrophilic and hydrophobic molecules [[32], [33], [34], [35]]. These platforms have shown efficacy in biomedical applications such as post-infarction vascular regeneration [36] and immunotherapeutic interventions like islet transplantation for type 1 diabetes [37]. However, the tunability of these systems in terms of materials and compartmental design demands strict consideration of biocompatibility, mechanical stability, and retention capacity. In addition, double emulsions can facilitate the self-assembly of phospholipids or synthetic polymers into membrane-like structures resembling liposomes or polymersomes [38,39]. Despite their utility in mimicking cellular architecture and enhancing drug transport, such constructs often lack the mechanical resilience needed to withstand gastric acidity and enzymatic degradation, thus posing limitations for oral drug delivery. To address this, recent developments have employed triple emulsion droplets featuring an intermediate oil layer situated between a hydrogel shell and a liquid core [40,41]. This design effectively separates the bioactive payload from the external environment, allowing for the protection and controlled release of encapsulated agents in response to specific stimuli [42,43]. However, bioactive compounds such as PTX, known for their anti-inflammatory and antioxidant properties, face considerable challenges in achieving long-term stability when exposed to oxidative environments. This limitation undermines the full potential of microfluidic-based drug delivery systems, particularly in applications requiring oral administration. In addition, the free radicals generated during photopolymerization—commonly employed to form the outer shell of hydrogel microcapsules—can further exacerbate oxidative degradation of sensitive compounds like PTX. Hence, the development of a microcapsule architecture that can effectively isolate PTX from oxidative stress while enabling controlled, stimulus-responsive release is crucial for preserving its therapeutic efficacy throughout gastrointestinal transit. Despite the promise of microfluidic encapsulation technologies, the feasibility and performance of PTX delivery using such systems remain largely unexplored and warrant further investigation.

In this study, we present a pH-responsive core–shell hydrogel microcapsule system designed to improve the oral delivery of PTX, thereby enhancing its bioavailability and therapeutic efficacy against IBD when compared to unencapsulated oral administration. These microcapsules are fabricated using a triple emulsion template followed by photopolymerization, forming a structure comprising a poly (acrylic acid)-poly (ethylene glycol) diacrylate (PAA–PEGDA) outer shell and a PEGDA core, with a thin intermediate oil layer in between. The interstitial oil layer plays a dual functional role: it acts as a diffusion barrier that promotes efficient drug encapsulation within the porous hydrogel matrix, and it establishes a localized pH-stable environment that maintains PTX activity until triggered release occurs under mildly basic gut conditions (pH 7.5) via osmotic swelling of the shell. This design not only improves encapsulation stability and loading efficiency, but also highlights the importance of oil-mediated compartmentalization in developing responsive drug delivery vehicles. The chemically crosslinked PEGDA-based hydrogel network also preserves the bioactivity of redox-sensitive compounds like PTX, offering a biocompatible and effective strategy for encapsulating labile therapeutic agents. As a result, the system effectively shields PTX from acidic degradation in the stomach, extends its residence time within the colon, and promotes localized accumulation. Collectively, this prolonged colonic retention enhances the therapeutic impact of PTX by targeting gut inflammation and microbiota dysregulation in the IBD setting.

## 2 Materials and methods

### 2.1 Materials

Mineral oil, soybean oil, poly (ethylene glycol) diacrylate (PEGDA, Mn 700 Da), poly (acrylic acid) (average M_v_ ∼4,000,000), poly (vinyl alcohol) (PVA, M_w_ 13,000–23,000, 87–89% hydrolyzed), erioglaucine disodium salt, *n*-octadecyltrimethoxysilane, Span® 80 (Sorbitan monooleate, viscosity 1000–2000 mPa s (20 °C), fluorescein isothiocyanate-dextran (FITC-DEX, Mw = 2,000,000), 2-Hydroxy-2-methylpropiophenone (photoinitiator), hydrochloric acid solution (1.0 N, BioReagent, suitable for cell culture), pentoxifylline (PTX), and 5-aminosalicylic acid (5-ASA) were purchased from Sigma-Aldrich (St. Louis, MO, USA). Polyglycerol polyricinoleate (PGPR) was provided by ILSHINWELLS Co. (Mapo-gu, Seoul, Korea). Fetal bovine serum (FBS) was obtained from GE Healthcare Life Sciences (Logan, UT, USA). The following antibodies were obtained: COX-2, iNOS, phospho-NF-κBp65, NF-κBp65 antibodies, β-actin antibody and macrophage-specific monoclonal antibody F4/80 (Santa Cruz Biotechnology, Paso Robles, CA, USA); HRP-conjugated goat anti-mouse and goat anti-rabbit IgG antibodies (Gene Tex, Irvine, CA, USA). Dextran sulfate sodium (M_w_ = 36,000 to 50,000) was obtained from MP Biomedicals (Santa Ana, CA, USA). All other reagents did not show any critical cytotoxic effects by themselves.

### 2.2 Fabrication of glass capillary microfluidic device and its operation

The fabrication of a glass capillary microfluidic device begins with the preparation of an injection capillary, derived from a circular glass tube with an initial inner diameter of 580 μm, which is tapered to achieve a final inner diameter of 100 μm. To impart hydrophobicity to the inner surface, the capillary is treated with trichloro (octadecyl) silane for 10 min, followed by rinsing with ethanol. The treated injection capillary is then inserted into a square capillary with an inner width of 1.05 mm, slightly larger than the injection capillary's outer diameter of 1 mm. A secondary tapered glass capillary, fabricated by drawing a cylindrical capillary to an outer diameter of 20 μm, is positioned inside the injection capillary to enable the co-flow of two immiscible fluids, serving as the innermost and inner phases. For the collection of the generated droplets, a collection capillary tapered to an inner diameter of 350 μm is employed, with its inner surface similarly rendered hydrophobic via trichloro (octadecyl) silane treatment. The generation of emulsion droplets is precisely regulated by syringe pumps (Legato100, KD Scientific, Holliston, MA, USA) controlling the volumetric flow rates. Droplet formation is observed and analyzed in real time using an inverted fluorescence microscope (Nikon Eclipse Ti2, Minato City, Tokyo, Japan) equipped with a high-speed camera (MINI UX 50, Minato City, Tokyo, Japan).

### 2.3 Characterization of hydrogel microcapsules

The hydrogel microcapsules were examined using an inverted fluorescence microscope (Nikon Eclipse Ti2) equipped with a sCMOS camera (Zyla, Andor Technology, Belfast, UK). Particle analysis was conducted using the software programs ImageJ (National Institute of Health) and NIS-Elements (Nikon). The temporal release profiles under external stimuli were observed and characterized using the microscope.

### 2.4 Quantitative analysis of *in vitro* release behavior from the microcapsules

The concentration of blue dye (Erioglaucine disodium salt) as a model active in each microcapsule suspension was quantified using a UV–Vis spectrophotometer (GENESYS 180, Thermo Scientific, Waltham, MA, USA) at room temperature. *In vitro* release experiments were conducted using PHM microcapsules identical to those employed in the *in vivo* studies, consisting of a PEGDA-based hydrogel core, a pH-responsive PAA–PEGDA hydrogel shell, and an interlayer oil phase. As a model compound, a hydrophilic dye, erioglaucine disodium salt (0.1 wt%), was dissolved in the innermost aqueous phase. Two sets of microcapsule suspensions containing an equal number of microcapsules were prepared and dispersed in either artificial gastric fluid (AGF, pH 2) or artificial intestinal fluid (AIF, pH 7.5). AGF was prepared as an acidic medium containing sodium chloride, pepsin, and hydrochloric acid, while AIF was prepared according to USP (United States Pharmacopeia) standard formulations based on potassium phosphate (KH_2_PO_4_) with pH adjustment using sodium hydroxide. Release under AIF conditions is attributed not to shell degradation but to pH-induced swelling of the PAA–PEGDA shell and the subsequent destabilization of the interlayer oil phase. The concentration of blue dye was determined by measuring the absorbance at its maximum wavelength (*λ*_max_ ≈ 630 nm). Measurements were performed in triplicate to ensure reproducibility and calculate error bars.

### 2.5 Cells culture

Human gastrointestinal epithelial Caco-2 cells, human hepatocellular carcinoma HepG2 cells, and murine macrophage RAW 264.7 cells were obtained from the American Type Culture Collection (ATCC; Manassas, VA, USA). Caco-2 and HepG2 cells were cultured in Dulbecco's Modified Eagle's Medium (DMEM; GE Healthcare, Logan, UT, USA), while RAW 264.7 cells were maintained in RPMI-1640 medium (GE Healthcare, Logan, UT, USA). All culture media were supplemented with 10% fetal bovine serum (FBS), 100 U/mL penicillin, and 100 μg/mL streptomycin. Cells were incubated at 37 °C in a humidified atmosphere containing 5% CO_2_. For experiments, cells were seeded onto culture plates at a density of 1 × 10^6^ cells/cm^2^ and allowed to reach confluence. Prior to treatment with PHM, cells were washed twice with phosphate-buffered saline (PBS) and serum-starved for 24 h in FBS-free DMEM or RPMI-1640, as appropriate. Inflammatory responses in RAW 264.7 cells were induced by stimulation with lipopolysaccharide (LPS, 1 μg/mL) for 24 h.

### 2.6 Cell viability assay

The viability assessment of Caco-2, HepG2, and RAW 264.7 cells was carried out utilizing the EZ-CYTOX kit (Dail-Lab Service, Guro-gu, Seoul, Korea) following the manufacturer's guidelines. The cells were cultured in 96-well culture plates, and subsequent to incubation with PHM, 10 μL of the EZ-CYTOX master mix was introduced into each well and allowed to incubate for 1 h. Cell viability was subsequently assessed directly employing a microplate reader (SPARK, Seestrasse, Männedorf, Switzerland) at a wavelength of 450 nm.

### 2.7 Experimental animals and induction of colitis

All animal procedures were conducted in accordance with the National Institutes of Health Guidelines for the Humane Treatment of Laboratory Animals and were approved by the Institutional Animal Care and Use Committee of Pukyong National University (Approval No. PKNUIACUC-2025-05). ICR mice (male), aged 7 weeks, were obtained from Hyochang Science (Seo-gu, Daegu, Korea). All mice were supplied water and a commercial diet ad libitum and kept for at least 7 days before the experiments. We utilized a DSS-induced murine model of IBD, which is a widely recognized preclinical system for investigating acute colitis, in accordance with protocols established in prior studies [44]. The experiment comprised eight groups, each containing five mice (n = 5). Group 1 (Control) received only regular drinking water without DSS exposure. Group 2 (IBD model) was given 4% DSS in 5 mL of drinking water per mouse daily for seven days to induce colitis. Group 3 (DSS + PTX) was treated with either 10 mg/kg or 100 mg/kg of PTX via oral gavage every other day for 14 days. Starting on day 7, these mice also received 4% DSS for seven days, following the same protocol as Group 2. Group 4 (DSS + PHM) received PHM microcapsules at a dose equivalent to 10 mg/kg (1 × 10^3^ capsules/mL) or 100 mg/kg (1 × 10^4^ capsules/mL) of PTX via oral gavage every other day for 14 days, with 4% DSS administered concurrently from day 7 to day 14, identical to the regimen in Group 2. Group 5 (DSS + empty microcapsules) was treated with empty microcapsules (1 × 10^4^ capsules/mL, lacking PTX) following the same oral administration and DSS exposure schedule as Group 4. Group 6 (DSS + 5-ASA) was administered 5-aminosalicylic acid (5-ASA) at a dose of 200 mg/kg via oral gavage every other day for 14 days, with 4% DSS treatment initiated on day 7 and maintained for seven days. Two additional groups received only single-agent treatments. Group 7 (PTX alone) was given PTX (100 mg/kg) via oral gavage every other day for 14 days, while Group 8 (PHM alone) received PHM (1 × 10^4^ capsules/mL) under the same schedule, without DSS co-administration. Throughout the *in vivo* study, mouse body weight was monitored every other day. Disease activity index (DAI) scores were assessed daily on a 0–4 scale, following the criteria outlined in Table S1 [45], and mean DAI values were calculated for each group. On day 15, fecal samples were collected and stored at −70 °C for subsequent gut microbiota profiling via 16S rRNA gene sequencing. Blood samples were also obtained for general toxicity assessment. Following sample collection, all animals were euthanized, and their colons and spleens were excised, rinsed, and measured for both length and weight. A 0.5 cm segment of the distal colon was dissected, embedded in O.C.T. compound (Sakura Finetek, CA, USA), snap-frozen at −70 °C, and cryosectioned into 7 μm slices using a cryostat. The sections were then mounted on SuperFrost Plus microscope slides (Thermo Fisher Scientific, IL, USA) for hematoxylin and eosin (H&E) staining and fluorescence-based immunohistochemical analysis. Histological damage was evaluated independently by two experienced gastrointestinal pathologists in a blinded manner, following previously published scoring criteria with minor modifications [46]. Briefly, H&E-stained sections were examined for mucosal ulceration, crypt epithelial damage, and inflammatory changes involving at least one-third of the mucosal layer. Scores for ulceration and injury were combined to produce a composite histological damage score. Additional portions of the distal colon were preserved at −70 °C for downstream molecular analyses, including RNA extraction for quantitative reverse transcription PCR (qRT-PCR) and protein isolation for Western blotting.

### 2.8 Periodic acid–schiff (PAS) staining

PAS staining was performed to assess mucosal integrity and goblet cell distribution in intestinal tissues. Frozen intestinal samples were sectioned using a cryostat, air-dried, and processed using a commercial PAS staining kit (Medilab, Guri, Gyeonggi-do, Korea) according to the manufacturer's instructions. Briefly, cryosections were rehydrated, oxidized with periodic acid solution, and incubated with Schiff's reagent to visualize polysaccharides and mucin-containing structures. Sections were subsequently counterstained with hematoxylin, rinsed with distilled water, and mounted using an aqueous mounting medium. PAS-stained sections were observed under a light microscope. Images were acquired using an Axioskop 2 plus microscope equipped with an AxioCam MRc5 CCD camera (Carl Zeiss, Jena, Thuringia, Germany).

### 2.9 General toxicity test

Blood was collected from the retro-orbital venous plexus of mice using heparin-coated capillary tubes (Kimble Chase, Vineland, NJ, USA). Serum was immediately isolated by centrifugation at 3000×*g* for 15 min at 4 °C. Biochemical parameters, including aspartate aminotransferase (AST), alanine aminotransferase (ALT), and lactate dehydrogenase (LDH), were quantified using an automated clinical chemistry analyzer (Hitachi 7180, High-Technologies Corp., Minato, Tokyo, Japan).

### 2.10 Fluorescence immunohistochemistry analysis

Distal colon tissue sections mounted on slides were fixed in 4% paraformaldehyde prepared in PBS for 10 min at room temperature, followed by permeabilization with 0.1% Triton X-100 in PBS for 5 min. To reduce nonspecific binding, the samples were blocked for 30 min at 25 °C with PBS containing 5% (v/v) normal goat serum (NGS). Tissues were then incubated overnight at 4 °C with a rat monoclonal *anti*-F4/80 antibody. After three rinses with PBS, the sections were treated with Alexa Fluor 488-conjugated goat anti-mouse IgM secondary antibody (Invitrogen, Carlsbad, CA, USA) and counterstained with 4’,6-diamidino-2-phenylindole (DAPI) in PBS containing 5% NGS for 2 h at 25 °C. Following a final series of PBS washes, slides were mounted and visualized using an Olympus FluoView™ 300 confocal microscope equipped with a 400 × magnification objective (Olympus Corporation, Hachioji, Tokyo, Japan).

### 2.11 qRT-PCR analysis

Total RNA was isolated using the NucleoSpin® RNA Plus kit (Macherey-Nagel, Bethlehem, PA, USA) following the manufacturer's instructions. Complementary DNA (cDNA) was synthesized from 1 μg of total RNA using the ReverTra Ace qPCR RT Master Mix kit (TOYOBO, Osaka, Japan). For quantitative real-time PCR analysis, 2 μL of the resulting cDNA was used to amplify target genes, including IL-1β, IL-6, TNF-α, and Toll-like receptors TLR-4, TLR-5, and TLR-9, using the specific primers listed in Table S2. Reactions were carried out on a LightCycler 96 System (Roche, Basel, Switzerland) with the AccuPower 2 × Greenstar qPCR Master Mix (Bioneer, Daejeon, Korea), following the manufacturer's protocol. Thermal cycling conditions consisted of an initial denaturation at 95 °C for 10 min, followed by 40 continuous cycles of denaturation at 95 °C for 20 s, and combined annealing/extension at 55 °C for 30 s with real-time fluorescence data acquisition. Melting curve analysis was performed immediately after amplification to verify specificity of the PCR products. Gene expression levels were normalized using β-actin as an internal control.

### 2.12 Western blot analysis

Approximately 30 mg of tissue was homogenized in RIPA lysis buffer (ATTO Corp., Tokyo, Japan) to extract total protein. Protein concentrations were quantified using the BCA Protein Assay Kit (Pierce, Rockford, IL, USA). Equal amounts of protein (20 μg/sample) were separated on 8–12% SDS-PAGE gels and subsequently transferred onto polyvinylidene fluoride (PVDF) membranes. Membranes were rinsed with TBST buffer [10 mM Tris-HCl (pH 7.6), 150 mM NaCl, and 0.05% Tween-20], blocked with 5% skim milk for 30 min at 25 °C, and incubated overnight at 4 °C with the appropriate primary antibodies. After two washes with TBST, membranes were incubated for 2 h with horseradish peroxidase (HRP)-conjugated secondary antibodies. Protein bands were visualized using an enhanced chemiluminescence (ECL) detection system (Amersham Pharmacia Biotech Inc., Piscataway, NJ, USA) and imaged with a Chemi Doc™ XRS + System (Bio-Rad, Hercules, CA, USA). The relative band intensities were quantified using Scion Image software (Beta 4.02; Scion Corp., Frederick, MD, USA).

### 2.13 Synthesis of fluorescent PTX-conjugated carbon dots (PTX-CDs)

Fluorescent pentoxifylline-conjugated carbon dots (PTX-CDs) were synthesized via a hydrothermal-assisted method, as described previously [47], with minor modifications. First, nitrogen- and phosphorus-co-doped carbon dots (NPCDs) were prepared by dissolving p-toluidine (0.1 g) and adenosine triphosphate (ATP, 0.1 g) in 10.0 mL of distilled water, followed by sonication for 30 min to ensure a homogeneous suspension. The resulting solution was transferred into a 50 mL Teflon-lined stainless-steel autoclave, and ethanol was added to fill 80% of the total volume. The autoclave was sealed and heated at 180 °C for 10 h. After cooling to room temperature, the resulting brown solution was centrifuged at 10,000×*g* for 10 min to remove large particles. The supernatant was dialyzed using 1000 Da dialysis tubing (Spectrum Chemical, New Brunswick, NJ, USA) for 12 h. To remove the remaining solvent and prevent particle aggregation, the solution was lyophilized using a freeze dryer (Sam Won, Seoul, Korea), yielding black-brown NPCD powders, which were stored at 4 °C for further use. To conjugate PTX, various concentrations of PTX stock solutions were prepared in distilled water. Then, 10 μL of NPCDs (10.0 μg/mL), 985 μL of pure water (pH 7.0), and 5 μL of PTX solution were mixed in a centrifuge tube. After incubation at room temperature for 1 min, the fluorescence intensity of PTX-CDs was measured using spectrophotometric analysis (excitation: 362–396 nm; emission: 432–480 nm). The fluorescent PTX concentration was quantified using a standard calibration curve. Additionally, to evaluate the biodistribution of PTX, a sufficient amount of PTX-CDs was lyophilized using a freeze dryer to obtain a powdered form suitable for long-term storage and subsequent *in vivo* application.

### 2.14 Biodistribution of PTX and PHM

To quantify PTX concentrations in gastrointestinal tissues, mice were orally administered either PTX-carbon dots (PTX-CDs, 100 mg/kg) or PHM encapsulating PTX-CDs (1 × 10^4^ capsules/mL, equivalent to 100 mg/kg PTX-CDs). Gastrointestinal tissues were collected at 1, 3, 6, and 12 h post-administration. After euthanasia, the stomach, small intestine, and colon were excised, thoroughly rinsed with saline to remove luminal contents, and homogenized. PTX concentrations in tissue homogenates were quantified by spectrophotometric analysis using excitation/emission wavelengths of 362–396 nm/432–480 nm. Absolute PTX levels were determined from standard calibration curves generated using known quantities of PTX-CDs and normalized to tissue weight (g). To evaluate the time-dependent gastrointestinal distribution of PHM, mice were orally administered PHM incorporated into the hydrogel shell with fluorescein isothiocyanate–dextran (FITC–DEX, Mw = 2,000,000) at a concentration of 1 × 10^4^ capsules/mL. Gastrointestinal samples were collected at 1, 3, 6, 12, and 24 h post-administration. Following euthanasia, the stomach, small intestine, colon, and feces were harvested, gently washed to remove residual contents, and homogenized. PHM distribution in each gastrointestinal segment and feces was quantified spectrophotometrically based on FITC fluorescence (excitation at 488 nm and emission at 520 nm) using standard calibration curves constructed from known capsule numbers. Results are expressed as the number of capsules per gastrointestinal segment.

### 2.15 16S rRNA gene sequencing of gut microbiota analysis

For microbiome analysis, total genomic DNA was extracted from frozen fecal samples using the Fast DNA® Spin Kit for Soil (MP Biomedicals, Solon, OH, USA) following the manufacturer's protocol. The V3–V4 regions of the bacterial 16S rRNA gene were amplified using specific primers listed in Table S3 [48]. The purified PCR products were then indexed with Illumina NexTera barcodes using the Nextera XT Index Kit (Illumina, San Diego, CA, USA). Barcoded amplicons were pooled and sequenced using the Illumina MiSeq platform (Illumina, San Diego, CA, USA), with sequencing services provided by ChunLab Inc. (Seocho-gu, Seoul, Korea). Taxonomic profiling and sequence analysis were performed using the EzBioCloud 16S-based Microbial Taxonomic Profiling platform (ChunLab Inc.), which enables comparative microbiota analysis. Taxonomic assignments were conducted using the curated ChunLab 16S rRNA database (DB ver. PKSSU4.0) [49]. OTU clustering was performed using CD-HIT and UCLUST algorithms at a 97% sequence similarity threshold [50,51]. Alpha diversity metrics, including Chao1 richness and Shannon diversity index, were calculated to assess microbial diversity within samples.

### 2.16 Statistical analysis

All data are presented as mean ± standard error (SE). Statistical analyses were performed using one-way analysis of variance (ANOVA), and when appropriate, post hoc comparisons between treatment groups and the control group were conducted using the Bonferroni-Dunn test. Analyses were carried out using SPSS software version 16.0 (IBM Corp., Armonk, NY, USA). A *p*-value of ≤0.05 was considered indicative of statistical significance.

## 3 Results and discussion

### 3.1 Production of PTX-loaded core-shell hydrogel microcapsules via triple emulsion-based microfluidics

**Fig. 1:**
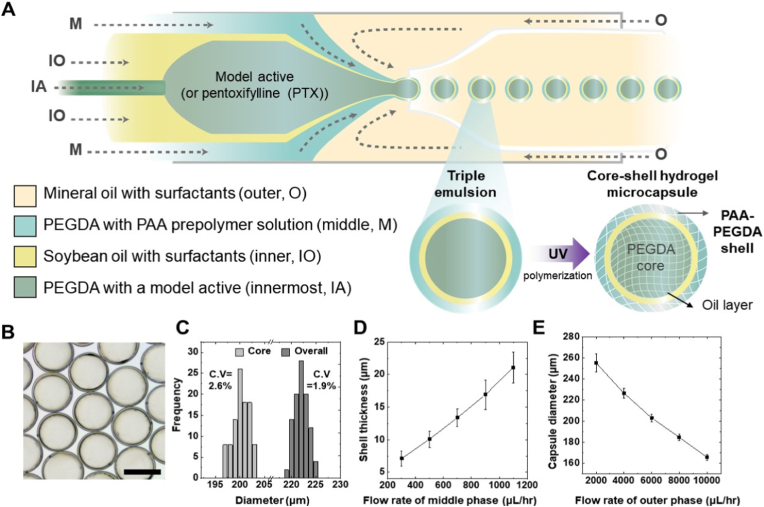
**Production of PTX-loaded core-shell hydrogel microcapsules via triple emulsion-based microfluidics. (**A) Schematic diagram illustrating the microfluidic setup to achieve reliable production of PAA–PEGDA-shelled microcapsules. (B) Optical micrograph of the as-prepared PTX-loaded capsules showing uniform spherical morphology. Scale bar represents 200 μm. (C) Graph showing the measured core and shell dimensions obtained from image analysis. (D) Graph of shell thickness as a function of the middle phase flow rate, demonstrating controlled modulation of the hydrogel barrier. (E) Graph of capsule diameter as a function of the continuous phase flow rate, confirming hydrodynamic regulation of capsule size.

We demonstrate that the triple emulsion-based microfluidic technique enables the consistent production of PTX-loaded core-shell hydrogel microcapsules, as shown in the schematic diagram of Fig. 1A. Triple emulsion droplets, serving as capsule templates, are prepared using a capillary microfluidic device, with the fabrication procedure described in detail in the Experimental section. To form triple emulsion drops, an aqueous hydrogel prepolymer solution (IA) is introduced through the smaller tapered capillary to form the innermost core; For instance, the innermost aqueous phase comprises a photopolymerizable aqueous solution of polyethylene glycol diacrylate (PEGDA, 10%) and PTX, or blue dye as a model active compound. An inner oil phase (soybean oil with 5 wt% polyglycerol polyricinoleate, IO) is introduced with the aqueous hydrogel prepolymer solution through the injection capillary. This coaxial biphasic stream formed in the injection capillary allows a periodic stream of hydrogel prepolymer drops in the oil phase due to the preferential wetting of the oil phase to the hydrophobically treated inner wall of the injection capillary. This flow behavior enables the formation of a thin lubricating oil layer between the innermost aqueous prepolymer drop and the hydrophobic wall of the injection capillary. Next, an additional hydrogel prepolymer solution (M) is introduced through the interstices between the injection capillary and the square capillary, consisting of 10% PEGDA, 2% polyvinyl alcohol (PVA), and 0.2% polyacrylic acid (PAA). The triphasic fluid stream is sheared by an outer oil phase (mineral oil with 2.5 wt% Span80, O) at the entrance of the collection capillary, forming uniform triple emulsion drops with a thin oil layer, as shown in Fig. 1A. These triple emulsion drops, consisting of photopolymerizable PEGDA for the middle and innermost core phases, are exposed to UV illumination, allowing the one-step formation of PEGDA-in-oil-in-PEGDA-PAA (PEGDA/O/PAA-PEGDA) core-shell hydrogel microcapsules (Fig. 1A). The resulting PHM are highly monodisperse, while compositions of each compartment are varied, demonstrating robustness and consistency of our proposed approach, as shown in optical micrographs of Fig. 1B; the size distribution plot shows that the addition of high-viscosity PAA has a negligible impact on particle size (Fig. 1C). The overall size and shell thickness of the microcapsule can be precisely controlled by adjusting the flow rates of the middle and outer phases, respectively. Specifically, increasing the flow rate of the middle phase results in a thicker shell, while varying the flow rate of the outer phase directly influences the overall size of the microcapsules, as demonstrated in Fig. 1D and E. To quantitatively evaluate the drug loading characteristics, a UV–Vis–detectable blue dye was used as a model compound, allowing reliable absorbance-based quantification at the microcapsule level. This approach was employed to assess both the loading amount per capsule and the encapsulation efficiency in a reproducible manner. A strong linear correlation was observed between the number of hydrogel microcapsules and the concentration of the released dye (Fig. S1A–B), demonstrating that the encapsulated amount per capsule is highly uniform and can be accurately quantified based on capsule number. Encapsulation efficiency was further evaluated by directly comparing the absorbance of the dye-containing emulsion prior to microcapsule formation with that measured from an equivalent number of hydrogel microcapsules after encapsulation (Fig. S2). The absorbance values under these two conditions were nearly identical, corresponding to an encapsulation efficiency of approximately 99.6%. Collectively, these results confirm that the triple emulsion–based microfluidic fabrication process enables highly reproducible encapsulation of active compounds with minimal loss during microcapsule formation.

### 3.2 pH-responsive hydrogel microcapsules for targeted drug delivery

Efficient targeted oral drug delivery systems should protect active compounds from degradation in the acidic gastric environment and enable controlled release in the intestine. To achieve this, PHM were designed with a protective oil layer between two hydrogel compartments and a pH-responsive PAA–PEGDA hydrogel shell. Given its crucial role in enabling pH-responsive swelling, we note that PAA is an anionic polyelectrolyte that exhibits a reversible conformational transition (coil-to-globule transition) starting around pH 5, driven by the gradual ionization of its carboxylic groups. Specifically, at this pH, initial swelling occurs as the ionization introduces electrostatic repulsion between COO^−^ groups and increases osmotic pressure within the polymer matrix. However, significant and visible swelling of the 3D polymer network becomes prominent at higher pH levels (e.g., pH 7 or above) as the degree of ionization and electrostatic interactions are maximized [52]. Therefore, we hypothesize that the pH-responsive swelling of the PAA–PEGDA shell compresses the oil layer radially, destabilizing it and enabling the pH-responsive release of the encapsulated cargo. To evaluate this mechanism, we encapsulated blue dye (model active) in the core compartment of the microcapsules and monitored its release behavior after exposure to acidic (pH 2) and basic (pH 7.5) environments. The release behavior was observed using optical microscopy, and osmolarity between the core compartment and the aqueous media was carefully matched to prevent water transport driven by osmotic pressure differences, which could otherwise affect the destabilization of the oil layer.

**Fig. 2:**
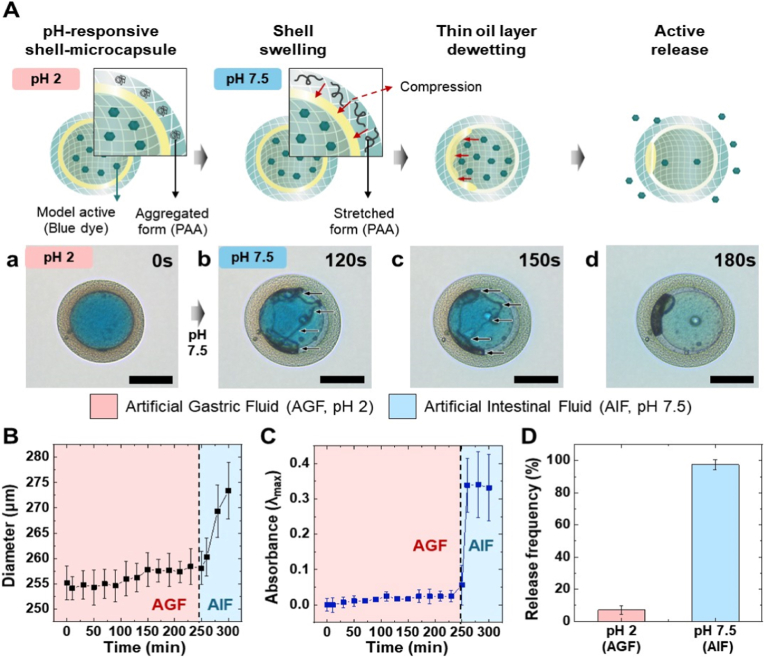
**pH-responsive hydrogel microcapsules for targeted drug delivery.** (A) Schematic illustration of the release mechanism showing pH-responsive swelling of the PAA–PEGDA shell and subsequent rupture of the oil layer, leading to active release. Optical micrographs of PTX-loaded capsules under acidic (pH 2, AGF) and basic (pH 7.5, AIF) conditions: (a) stable morphology at pH 2, (b) initiation of oil layer rupture at 120 s, (c) progression of rupture at 150 s, and (d) complete rupture and dye release at 180 s. Scale bars, 100 μm. (B) Time-dependent diameter changes of the capsules in AGF (pH 2) followed by AIF (pH 7.5). (C) Absorbance measurements of the encapsulated dye showing negligible release in AGF and rapid release in AIF. (D) Release frequency of capsules under acidic and basic conditions, confirming structural stability in AGF and selective release in AIF.

Under acidic conditions (pH 2), the microcapsules exhibit high structural stability, as demonstrated in Fig. 2A–a, where no rupture or deformation of the oil layer is observed. However, when exposed to a basic condition (pH 7.5), the PAA–PEGDA shell undergoes spatial swelling, analogous to the behavior expected in the intestinal environment, exerting radial compression on the oil layer. This process initiates oil rupture within 120 s (Fig. 2A–b), with complete rupture occurring within 180 s, thereby enabling the release of the encapsulated dye (Fig. 2A–c, d). The ruptures of the oil layer are observed through morphological changes at the oil-water interface, which are denoted by black arrows. This unique release pattern is attributed to the selective swelling of the shell. The presence of the hydrogel core imposes greater pressure on the oil layer between the two compartments when the shell is compressed, thereby facilitating its rupture.

To investigate the release pattern in detail, we quantitatively analyzed the changes in the overall diameter of the microcapsules and correlated this with the release behavior using absorbance measurements of the encapsulated dye (Fig. 2B and C). In this experiment, the capsules were observed in artificial gastric fluid for 4 h and subsequently transferred to simulated intestinal fluid for further observation. This stability aligns with the typical residence time of food or drugs in the stomach, approximately 4 h, ensuring adequate protection throughout gastric transit [53]. Fig. 2B shows that the microcapsules exhibit negligible diameter changes in artificial gastric fluid (AGF), whereas dramatic diameter changes occur in artificial intestinal fluid (AIF). These results are consistent with the absorbance of the encapsulated dye, demonstrating that the release occurs due to the capsule diameter changes induced by shell swelling (Fig. 2C). To quantitatively evaluate the release behavior under AIF conditions, the time-dependent release data were fitted to the Korsmeyer–Peppas model (Fig. S3). The analysis yielded a release exponent of n = 1.31 (R^2^ ≈ 0.94), indicating release characteristics distinct from simple diffusion. These kinetic results suggest that pH-responsive shell swelling and the subsequent destabilization of the oil layer are closely associated with the release behavior observed under intestinal-mimicking conditions. Fig. 2D presents the statistics of capsules undergoing release, demonstrating that the proposed microcapsules remain highly stable in artificial gastric fluid while enabling selective release in artificial intestinal fluid. Taken together, these results demonstrate that the proposed microcapsules can serve as a highly efficient intestinal-targeted delivery system.

### 3.3 Enhanced colonic targeting and sustained release of PTX via core–shell hydrogel microcapsules

**Fig. 3:**
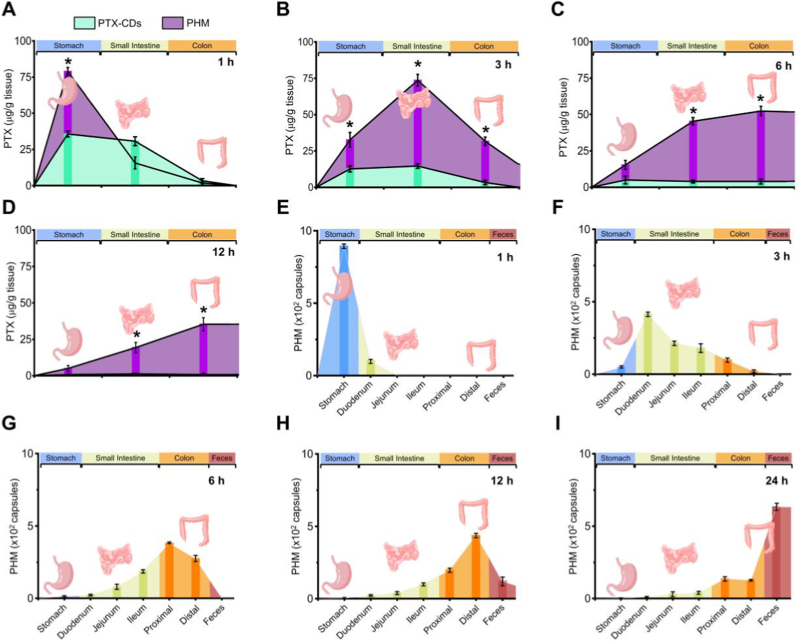
**Biodistribution of PTX and PHM.** Gastrointestinal distribution of PTX in ICR mice following oral administration of either PTX–carbon dots (PTX-CDs, 100 mg/kg) or PHM encapsulating PTX-CDs (1 × 10^4^ capsules/mL, equivalent to 100 mg/kg PTX-CDs), assessed at 1 h (A), 3 h (B), 6 h (C), and 12 h (D). PTX concentrations in tissue homogenates were quantified by spectrophotometric analysis using excitation/emission wavelengths of 362–396 nm/432–480 nm ∗*p* ≤ 0.05 vs PTX-CDs. Data are shown as mean ± SE (n = 5). Time-dependent gastrointestinal distribution and clearance of PHM following oral administration of PHM incorporated into the hydrogel shell with fluorescein isothiocyanate–dextran (FITC–DEX, Mw = 2,000,000) at a concentration of 1 × 10^4^ capsules/mL. Fluorescence signals were quantified in the stomach, small intestine, colon, and feces at 1 h (E), 3 h (F), 6 h (G), 12 h (H), and 24 h (I) post-administration using FITC fluorescence (excitation at 488 nm and emission at 520 nm). Data are presented as mean ± SE (n = 5).

To evaluate the gastrointestinal distribution of orally administered PTX, mice received either PTX-derived carbon dots (PTX-CDs, 100 mg/kg) or PTX-CDs encapsulated within hydrogel microcapsules (PHM, 1 × 10^4^ capsules/mL, equivalent to 100 mg/kg PTX). PTX concentrations in gastrointestinal tissues were quantified at 1, 3, 6, and 12 h post-administration (Fig. 3). In the group administered free PTX-CDs, PTX levels peaked at 1 h in the stomach (36 ± 2.5 μg/g tissue) and small intestine (31 ± 3.1 μg/g), followed by a rapid decline by 6 h and near-complete clearance by 12 h (Fig. 3A–C). This distribution pattern reflects the fast absorption and systemic elimination typical of free PTX formulations. In contrast, the PHM group exhibited a prolonged and progressively shifting distribution. At 1 h, PTX predominantly accumulated in the stomach (80 ± 2.6 μg/g), where the microcapsules remained largely intact. Over time, PTX was gradually released as the capsules transited along the gastrointestinal tract, leading to elevated concentrations at 3 h in the small intestine (74 ± 4.0 μg/g) and a marked accumulation by 6 h in the colon (52 ± 2.1 μg/g). At 12 h, PTX levels in the colon remained significantly high (35 ± 4.6 μg/g), particularly in the distal region, while free PTX had already been eliminated from all compartments (Fig. 3D). These findings indicate that PHM prolongs gastrointestinal residence time and facilitates colonic delivery of PTX. The spatiotemporal shift in PTX distribution aligns with intestinal transit kinetics, supporting the controlled release and mucosal retention capacity of PHM. Such features may enhance therapeutic potential for localized treatment of colonic inflammation and microbiota-related disorders. In contrast to the rapid clearance of free PTX, the PHM platform offers a non-invasive and targeted delivery strategy to improve PTX bioavailability and efficacy in gastrointestinal disease contexts.

Regarding the *in vivo* degradation behavior, PHM are not expected to undergo rapid biodegradation during gastrointestinal transit because the shell and core are formed by a chemically crosslinked PEGDA-based hydrogel network, which is known to be highly stable over short time scales. PEGDA undergoes UV-initiated free-radical polymerization to form a densely crosslinked three-dimensional matrix, and the resulting carbon–carbon backbone and ester crosslinks generally exhibit negligible hydrolysis within the limited residence time of the gastrointestinal tract (≤24 h). In this system, PAA is incorporated at a low content (0.2%) to impart pH-responsive ionization and swelling behavior rather than to introduce biodegradable linkages. Consequently, any structural changes occurring *in vivo* are expected to arise primarily from reversible pH-induced swelling/shrinkage and possible erosion-like surface alterations, rather than from true polymer cleavage or rapid mass loss. To experimentally validate these design-based expectations, we conducted an additional study to assess the *in vivo* behavior of PHM during gastrointestinal transit using a non-absorbable fluorescent tracer. Specifically, FITC–DEX was incorporated into the hydrogel shell of PHM to enable time-dependent evaluation of gastrointestinal distribution. Mice were orally administered PHM containing FITC–DEX at a concentration of 1 × 10^4^ capsules/mL, and gastrointestinal tissues were collected at 1, 3, 6, 12, and 24 h post-administration to quantify microcapsule distribution (Fig. 3E and F). At 1 h, the majority of the fluorescence signal was detected in the stomach (9.3 ± 0.2 × 10^2^ capsules), with minimal signal observed in the small intestine (Fig. 3E), indicating predominant gastric localization during the early post-administration phase. At 3 h, the fluorescence signal progressively shifted toward the small intestine, with increased accumulation in the duodenum (4.2 ± 0.1 × 10^2^ capsules), jejunum (2.2 ± 0.1 × 10^2^ capsules), and ileum (1.9 ± 0.2 × 10^2^ capsules), along with initial appearance in the proximal colon, reflecting forward gastrointestinal transit of PHM (Fig. 3F). Between 6 and 12 h, fluorescence signals became predominantly localized within the colon, particularly in the proximal and distal regions, with maximal accumulation observed in the distal colon at 12 h (4.4 ± 0.1 × 10^2^ capsules), while signals in the small intestine further declined. This distribution pattern indicates preferential colonic accumulation and sustained residence of PHM during this time window (Fig. 3G–H). By 24 h, a substantial portion of the fluorescence signal was detected in feces (6.5 ± 0.2 × 10^2^ capsules), reflecting progressive elimination of PHM from the gastrointestinal tract, although a measurable fraction of signal remained within the colon and small intestine (Fig. 3I). Collectively, these findings demonstrate that PHM exhibit a well-defined gastrointestinal transit and clearance profile over a 0–24 h period, with fluorescence signals largely confined to the intestinal lumen and predominantly eliminated through fecal excretion.

Given their microscale size (∼220 μm) and lack of systemic translocation, this distribution behavior further supports the favorable safety profile of PHM. Moreover, PHM exhibited no detectable cytotoxicity toward human gastrointestinal epithelial Caco-2 cells, human epithelial hepatocellular HepG2 cells, or murine macrophage RAW 264.7 cells across the tested concentration range over 24 h (Fig. S4A–B). In addition, PHM at 1 × 10^4^ capsules/mL attenuated lipopolysaccharide (LPS, 1 μg/mL)-induced IL-1β mRNA expression in RAW 264.7 cells, suggesting a potential anti-inflammatory effect under inflammatory conditions (Fig. S4C). Importantly, PHM alone did not induce pro-inflammatory cytokine expression in immune cells, indicating minimal immunogenicity. Collectively, these findings support the biocompatibility of PHM and further validate their safety for oral administration.

### 3.4 Therapeutic efficacy of PHM in mitigating symptoms of IBD

**Fig. 4:**
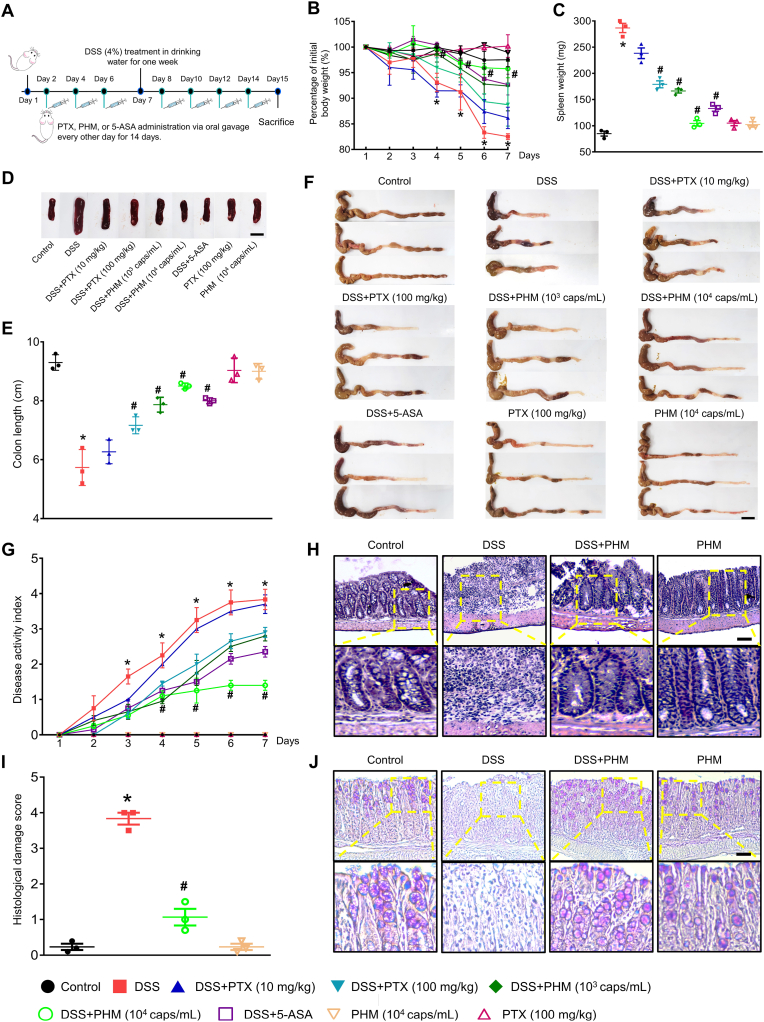
**Therapeutic efficacy of PHM in mitigating symptoms of IBD.** (A) ICR male mice (n = 5) were orally administered PTX (10 or 100 mg/kg), PHM (10^3^ or 10^4^ capsules/mL, equivalent to 10 or 100 mg/kg of PTX), or the standard IBD drug 5-ASA (200 mg/kg) every other day for 14 days. Starting on day 7, 4% DSS was introduced into the drinking water for 7 days to induce colitis. Control mice received regular drinking water without DSS. (B–C) Changes in body and spleen weights were measured. ∗*p* ≤ 0.05 vs Control, #*p* ≤ 0.05 vs DSS. n = 5. (D) Representative images of spleen are shown. Scale bar, 1 cm. (E) The effects of PHM on colon length are shown. ∗*p* ≤ 0.01 vs Control, #*p* ≤ 0.01 vs DSS. n = 3. (F) Representative images of colon are shown. Scale bar, 1 cm. (G) Disease activity index (DAI) scores were recorded during the experimental period. ∗*p* ≤ 0.01 vs Control, #*p* ≤ 0.05 vs DSS. n = 5. (H) Representative H&E-stained colon tissue sections are shown. Scale bar, 100 μm (magnification, × 100). (I) Histological damage scores were evaluated. ∗*p* ≤ 0.01 vs Control, #*p* < 0.01 vs DSS. n = 3. (J) Representative PAS-stained colon tissue sections are shown. Scale bar, 100 μm (magnification, × 100).

To investigate the preventive efficacy of PHM in attenuating intestinal inflammation and microbiota imbalance, we employed a DSS-induced IBD mouse model. DSS, a sulfated polysaccharide with high water solubility, is widely used to experimentally induce acute ulcerative colitis [54]. Its administration disrupts the intestinal epithelial barrier, leading to increased mucosal permeability and triggering osmotic stress that culminates in severe colonic inflammation. This is typically manifested by symptoms such as significant body weight loss, mucosal ulceration, colon shortening, and rectal bleeding, thereby recapitulating key pathological features of human ulcerative colitis [[54], [55], [56]]. To evaluate anti-inflammatory efficacy, mice were orally administered PTX (10 or 100 mg/kg), PHM (10^3^ or 10^4^ capsules/mL, equivalent to 10 or 100 mg/kg of PTX), or the standard IBD drug 5-ASA (200 mg/kg) every other day for 14 days (Fig. 4A). DSS exposure (4% in drinking water) was initiated on day 7 and continued for 7 days to induce acute colitis. In separate single-treatment groups, PTX (100 mg/kg) or PHM (10^4^ capsules/mL) was administered on the same alternate-day schedule for 14 days without DSS. Control mice received normal drinking water without DSS throughout the experiment. Following seven days of 4% DSS exposure, mice exhibited a 17.5% reduction in body weight, indicative of acute colitis development. Treatment with PTX at 100 mg/kg resulted in a moderate recovery, with a 7.1% increase in body weight compared to DSS-only controls (*p* ≤ 0.05, Fig. 4B). Notably, administration of PHM containing PTX led to greater weight restoration, with increases of 10.3% and 14.2% observed in the groups receiving 10^3^ or 10^4^ capsules/mL (equivalent to 10 and 100 mg/kg of PTX), respectively (*p* ≤ 0.05, Fig. 4B). The protective effect conferred by PHM at 10^3^ capsules/mL or higher was comparable to that of the standard treatment, 5-ASA.

Compared to the DSS-only colitis model, co-administration with either free PTX (100 mg/kg) or PHM (10^3^ or 10^4^ capsules/mL, equivalent to 10 or 100 mg/kg PTX) significantly mitigated colitis-related symptoms. Both treatment groups demonstrated reductions in spleen weight and size (*p* ≤ 0.05; Fig. 4C and D), preservation of colon length (*p* ≤ 0.01; Fig. 4E and F), and improvement in the disease activity index (DAI), which accounts for weight loss, diarrhea, and occult blood (*p* ≤ 0.05; Fig. 4G). Notably, PHM at 10^4^ capsules/mL exhibited the most pronounced protective effect, nearly abolishing DSS-induced symptoms. Even at the lower dose (10^3^ capsules/mL, equivalent to 10 mg/kg PTX), PHM achieved outcomes comparable to those observed with 5-ASA (200 mg/kg), a widely used therapeutic for IBD. In contrast, administration of PTX or PHM alone, in the absence of DSS-induced colitis, did not result in significant changes in body weight, spleen weight or size, colon length, or DAI, indicating that the protective effects observed were specific to the inflammatory condition rather than baseline physiological regulation. Histological analysis further supported these results. Colon tissues from PHM-treated mice (10^4^ capsules/mL) exhibited preserved crypt architecture, intact mucosa, and reduced inflammatory cell infiltration, as shown by H&E staining (Fig. 4H). Corresponding histological scores were significantly lower compared to DSS-only animals (*p* ≤ 0.01; Fig. 4I). Moreover, the PAS staining results demonstrate that DSS treatment markedly reduced PAS-positive goblet cells and disrupted the mucus layer, whereas treatment with PHM significantly restored goblet cell abundance and mucosal architecture (Fig. 4J). These findings complement the H&E histological analysis and provide additional evidence that PHM not only attenuates inflammation but also promotes recovery of the mucosal barrier. Taken together, these findings suggest that PHM enhances the local availability and mucosal residency of PTX in the colon, allowing for sustained anti-inflammatory effects with a lower systemic dose. This benefit is likely due to the protective hydrogel matrix, which delays drug release and aligns with gastrointestinal transit kinetics. While not dramatically superior in every parameter, the dose-sparing effect and tissue selectivity of PHM provide meaningful advantages over free PTX administration. Thus, PHM may represent a practical strategy to improve the therapeutic index of PTX in intestinal inflammatory conditions. For chronic inflammatory conditions, PTX has been clinically explored at daily doses ranging from 400 to 1200 mg in adults, depending on the indication, body weight, and presence of comorbidities [16,57]. Although interspecies dose translation is inherently complex due to physiological and metabolic differences, the body surface area (BSA)-based normalization method remains a widely accepted approach for estimating human equivalent doses (HED) in preclinical research. Based on the FDA-recommended Km values (Km = 3 for mice, 37 for humans), the standard mouse-to-human dose conversion factor is 12.3 [58]. Accordingly, the 100 mg/kg dose used in mice corresponds to an HED of approximately 8.13 mg/kg (i.e., 100 mg/kg ÷ 12.3). For an average adult weighing 60–70 kg, this translates to a daily dose of 488–569 mg, which is well within the clinically prescribed range for PTX (400–1200 mg/day). This dose calculation supports the translational appropriateness of our murine dosing regimen. Furthermore, when PTX is encapsulated in PHM, we observed comparable or superior therapeutic efficacy even at 10 mg/kg. This dose-sparing effect is likely due to enhanced colonic targeting, prolonged gastrointestinal residence time, and improved local bioavailability offered by the encapsulation system.

### 3.5 PHM mitigates colonic inflammation in a DSS-induced mouse model of IBD

**Fig. 5:**
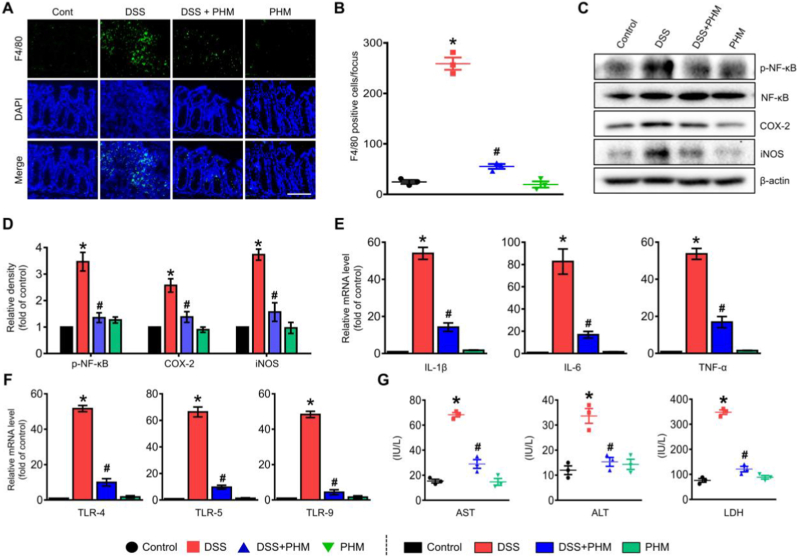
Therapeutic efficacy of PHM in mitigating symptoms of IBD. (A) Colonic expression of pro-inflammatory F4/80^+^ macrophages was visualized using confocal microscopy (green), with nuclear staining performed using DAPI (blue). Scale bar,100 μm (magnification × 200). n = 3. (B) Quantification of F4/80^+^ macrophages per field is presented. ∗*p* ≤ 0.01 vs Control, #*p* ≤ 0.01 vs DSS. n = 3. (C–D) Protein expression levels of phosphorylated NF-κB (p-NF-κB), COX-2, and iNOS were assessed to evaluate inflammatory signaling. ∗*p* ≤ 0.01 vs Control, #*p* ≤ 0.01 vs DSS. n = 5. (*E*–F) The levels of pro-inflammatory cytokines and Toll-like receptors (TLRs) were measured to determine the modulatory effects of PHM. ∗*p* ≤ 0.001 vs Control, #*p* ≤ 0.01 vs DSS. n = 5. (G) Serum levels of tissue injury markers (AST, ALT, and LDH) are shown to evaluate systemic toxicity. ∗*p* ≤ 0.001 vs Control, #*p* ≤ 0.01 vs DSS. n = 3. (For interpretation of the references to colour in this figure legend, the reader is referred to the Web version of this article.)

To further elucidate the mechanism by which PHM mitigates intestinal inflammation in DSS-induced colitis, we examined the infiltration of inflammatory macrophages within the colon. Immunofluorescence analysis revealed a pronounced accumulation of F4/80^+^ macrophages in the mucosal and submucosal layers of the distal colon in DSS-treated mice. In contrast, PHM-treated mice exhibited significantly reduced macrophage infiltration (*p* < 0.01, Fig. 5A and B), indicating a dampening of local immune activation. This suggests that PHM may prevent aberrant macrophage activation and reduce the translocation of luminal antigens into the intestinal mucosa, thereby attenuating the downstream inflammatory cascade. Such immunomodulatory effects may also contribute to reshaping the gut microbiota composition, as previously described [59].

The pathogenesis of IBD involves a complex interplay between innate immune cells, including macrophages and neutrophils, and dysregulation of the mucosal immune system, which comprises epithelial cells and T lymphocytes. Among the key regulators of this inflammatory process is the transcription factor nuclear factor-κB (NF-κB), which orchestrates the expression of numerous pro-inflammatory genes [60]. In IBD patients, aberrant activation of NF-κB—marked by its phosphorylation—has been observed in both epithelial cells and lamina propria lymphocytes, driving the transcription of cytokines such as IL-1β, IL-6, TNF-α, and the pro-inflammatory enzyme cyclooxygenase-2 (COX-2) [61,62]. Moreover, NF-κB signaling is critical for the differentiation and activation of inflammatory T helper cell subsets, including Th1 and Th17 cells [60]. To explore the potential regulatory effects of PHM on these inflammatory pathways, we assessed the phosphorylation levels of NF-κB, as well as protein expression of COX-2 and inducible nitric oxide synthase (iNOS) in colonic tissues by western blot analysis. COX-2 catalyzes the formation of prostaglandins, key mediators of inflammation and common targets of anti-inflammatory drugs [63]. iNOS, on the other hand, contributes to nitric oxide production, which is known to impair epithelial barrier integrity and exacerbate intestinal inflammation [64]. Our results demonstrated that oral administration of PHM significantly suppressed NF-κB phosphorylation and downregulated the expression of COX-2 and iNOS in the inflamed colon (*p* ≤ 0.01; Fig. 5C and D). These findings suggest that PHM may help attenuate intestinal inflammation by modulating molecular pathways associated with immune activation and integrity of the mucosal immune system within the colonic epithelium, indicating its potential utility as a supportive strategy in IBD management.

To further assess the anti-inflammatory effects of PHM, we conducted qPCR analysis on colon tissue to evaluate the expression of key inflammatory genes. Compared to DSS treatment alone, mice treated with DSS + PHM exhibited significantly reduced mRNA levels of pro-inflammatory cytokines, including IL-1β, IL-6, and TNF-α (*p* ≤ 0.01, Fig. 5E). These findings are consistent with previous studies demonstrating that the expression of these inflammatory genes is predominantly driven by the activation of innate immune cells, which subsequently promote Th1 and Th17 cell-mediated immune responses [65]. These results imply that PHM may contribute to preserving mucosal immune homeostasis in the inflamed colon. Beyond quantifying inflammatory cytokine transcripts, we examined toll-like receptors (TLRs), which are known to mediate immune recognition of microbial components and have been implicated in IBD pathogenesis [66]. Notably, DSS administration elevated the mRNA expression of TLR-4, -5, and -9, whereas PHM administration significantly suppressed their expression (*p* ≤ 0.01, Fig. 5F). This evidence corresponds with earlier studies that propose a firm relationship between the expression levels of TLR-4, TLR-5, and TLR-9 in IBD and the levels of IL-1β, IL-6, and TNF-α mRNA, these being associated with the endoscopic and histological manifestations of the disease [66,67]. While the precise role of TLRs in inflammation and microbial dysbiosis remains under investigation, our data support the potential involvement of PHM in modulating innate immune signaling. To evaluate systemic responses to DSS-induced tissue injury, we measured serum levels of aspartate aminotransferase (AST) and alanine aminotransferase (ALT), which indicate liver stress, as well as lactate dehydrogenase (LDH), a general marker of cellular damage. These markers were significantly elevated following DSS treatment, reflecting systemic toxicity. However, mice receiving oral PHM showed notably reduced levels of AST, ALT, and LDH (*p* ≤ 0.01, Fig. 5G), indicating that PHM may confer partial protection against DSS-induced systemic injury. These results indicate that, beyond its local anti-inflammatory activity in the gut, PHM may also help prevent secondary liver damage and systemic complications associated with IBD-related inflammation. Administration of PHM alone, under non-colitic conditions, did not result in significant changes in macrophage infiltration, pro-inflammatory protein and cytokine expression, TLR expression, or serum biochemical markers of tissue injury, indicating that PHM itself does not elicit inflammatory or toxic responses under physiological conditions. In line with our earlier findings showing a moderate recovery in DAI following PTX treatment (100 mg/kg), PTX administration in DSS-treated mice led to reduced pro-inflammatory cytokine levels and serum markers of tissue injury (Fig. S5A–B). In contrast, PTX alone did not affect these parameters under non-colitic conditions, suggesting **that its therapeutic activity is dependent on the inflammatory context.** Moreover, administration of empty microcapsules (PEGDA–PAA core–shell hydrogel microcapsules without PTX) did not produce significant changes in DAI (Fig. S6A), histological damage scores (Fig. S6B), or pro-inflammatory cytokine levels (Fig. S6C), compared with DSS-treated controls. In addition, no significant differences were observed in systemic serum toxicity markers (Fig. S6D), indicating the absence of detectable off-target or systemic adverse effects. Importantly, empty microcapsules alone also did not exert any therapeutic or aggravating effects on the evaluated parameters. These results confirm that the observed therapeutic benefits in the PTX-loaded PHM are attributable to the delivered drug rather than to the PEGDA–PAA microcapsule matrix itself.

Regarding safety, off-target effects, and systemic exposure, the PHM-based delivery strategy offers a rational approach to improving the therapeutic index of PTX. Conventional oral PTX administration is associated with rapid systemic absorption and broad tissue distribution, which can necessitate higher or repeated dosing to achieve efficacy in IBD and thereby increase the risk of dose-dependent off-target effects. In contrast, PHM are designed with a microscale hydrogel architecture that restricts intact carriers to the gastrointestinal lumen, preventing translocation across the intestinal epithelium. Consistent with this design, gastrointestinal distribution studies demonstrate lumen-confined localization and progressive fecal clearance, suggesting minimal systemic exposure of both the carrier and the encapsulated drug. In addition, pH-responsive PTX release from PHM under mildly alkaline conditions characteristic of the distal intestine and colon promotes localized drug availability at inflamed sites while limiting premature release in the upper gastrointestinal tract. This spatially controlled release, together with prolonged colonic residence, is expected to reduce systemic absorption and mitigate off-target effects. Importantly, therapeutic efficacy was achieved at a reduced PTX dose (10 mg/kg), further supporting the notion that PHM-mediated delivery enhances local bioavailability without increasing systemic burden. From a safety standpoint, PHM-treated animals exhibited no abnormal changes in body weight, gross behavior, or systemic toxicity markers, indicating good tolerability of the delivery system. Taken together, although future pharmacokinetic studies are warranted to quantitatively assess systemic PTX exposure, the present findings suggest that PHM-based delivery improves the safety margin of PTX by modulating its biodistribution, supporting both translational relevance and a favorable safety profile.

### 3.6 PHM helps balance and enhance the composition of the gut microbiota

**Fig. 6:**
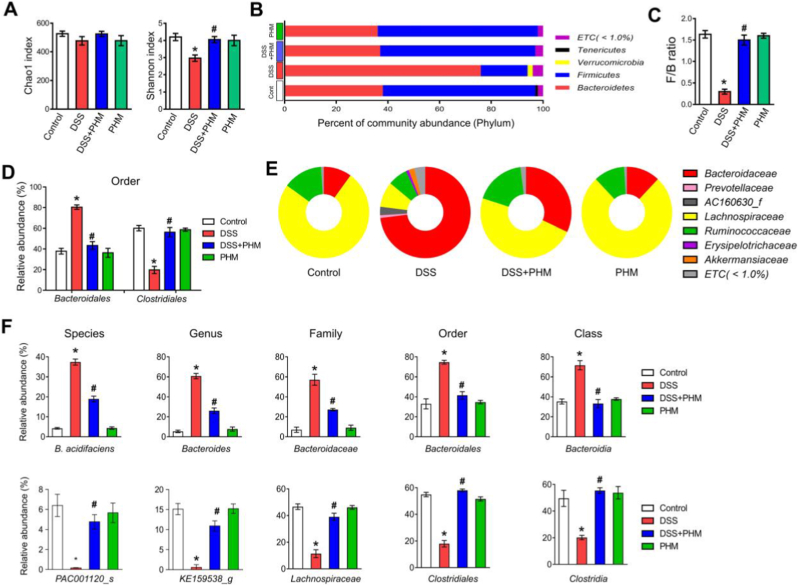
PHM helps balance and enhance the composition of the gut microbiota. (A) Microbial alpha-diversity and richness were evaluated using the Shannon and Chao1 indices, respectively. ∗*p* ≤ 0.05 vs. Control, #*p* ≤ 0.05 vs. DSS. n = 3. (B) The percentages of community abundance at the phylum level are shown as column diagrams. (C) The *Firmicutes*-to-*Bacteroidetes* (F/B) ratio is shown for DSS-treated mice with or without PHM administration. ∗*p* ≤ 0.01 vs. Control, #*p* ≤ 0.01 vs. DSS. n = 3. (D) Bar plots show the relative abundances of microbial taxa at the order level. ∗*p* ≤ 0.01 vs. Control, #*p* ≤ 0.01 vs. DSS. n = 3. (E) The family-level taxonomic composition of the gut microbiota is displayed using pie charts. (F) Boxplots show gut taxonomic communities of 2 dominant species (*Bacteroides acidifaciens and PAC001120*) in each sample. ∗*p* ≤ 0.01 vs Control, #*p* ≤ 0.01 vs DSS. n = 3. ∗*p* ≤ 0.01 vs. Control, #*p* ≤ 0.01 vs. DSS. n = 3.

Given the pivotal role of gut microbiota–mucosal immune interactions in the pathogenesis of IBD [68], we examined whether the therapeutic effects of PHM are linked to alterations in gut microbial composition. To this end, 16S rRNA gene sequencing was performed on fecal samples from four experimental groups (Vehicle, DSS + Vehicle, DSS + PHM, and PHM), and α-diversity was assessed to estimate microbial richness and taxonomic variety (Fig. 6). The Shannon index, which reflects microbial evenness and community diversity, showed no significant differences among groups, possibly due to the short treatment duration or the subtle nature of changes in overall microbial structure. In contrast, the Chao1 index, a measure of species richness, was significantly reduced in DSS-treated mice, indicating a marked loss of microbial taxa following colitis induction. Importantly, PHM treatment significantly restored the Chao1 index (*p* ≤ 0.05, Fig. 6A), suggesting that PHM may promote the recovery of gut microbial richness disrupted by DSS-induced inflammation. Restoration of microbial richness has been associated with improved metabolic flexibility and resilience of the gut ecosystem, which are critical for maintaining mucosal immune homeostasis [69]. This restoration of microbial richness may underlie the protective effects of PHM on intestinal immune homeostasis, and a prolonged pretreatment period may help optimize these microbiota-mediated benefits. To our knowledge, this is the first report demonstrating that PTX, when delivered via PHM, can influence microbiome diversity in an IBD model. While direct evidence linking PTX to microbiota modulation has been lacking, our findings are consistent with previous research showing that anti-inflammatory interventions can indirectly promote the restoration of gut microbial balance [68]. Therefore, it is plausible that the anti-inflammatory effects of PHM contribute to the normalization of microbial communities under colitic conditions. Moreover, both DSS and PHM treatments resulted in significant shifts the relative compositions of the gut microbiota community.

At the phylum level, DSS exposure led to an increased proportion of *Bacteroidetes* along with a marked reduction in *Firmicutes* compared to the Control group (Fig. 6B and C). This microbial imbalance reflects a reduced *Firmicutes*-to-*Bacteroidetes* (F/B) ratio, a dysbiosis pattern frequently observed in patients with IBD [70]. Interestingly, mice receiving PHM exhibited a partial restoration of the F/B ratio despite DSS challenge, suggesting that PHM may aid in mitigating dysbiosis by promoting compositional rebalancing of the gut microbiota (*p* ≤ 0.01, Fig. 6B and C). Within the *Firmicutes* phylum, the *Lachnospiraceae* family is known to contribute to carbohydrate metabolism and the production of butyrate and other short-chain fatty acids (SCFAs), which play a supportive role in maintaining mucosal immune function through interactions with gut microbes [71]. SCFAs, particularly butyrate, serve as a primary energy source for colonocytes and are known to enhance epithelial barrier integrity while suppressing excessive inflammatory signaling [71]. Conversely, the expansion of the *Bacteroidaceae* family, a part of *Bacteroidetes* phylum, has been frequently reported during gut inflammation [72]. In our study, PHM-treated mice showed a significant decrease in the abundance of *Bacteroidaceae* belonging to the *Bacteroidales* order and a concomitant increase in *Lachnospiraceae,* which belongs to the *Clostridiales* orders compared to DSS-treated mice (*p* < 0.05, Fig. 6D and E). Members of the *Lachnospiraceae* family are metabolically linked to carbohydrate fermentation pathways, including glycolysis and acetyl-CoA–dependent butyrate biosynthesis, suggesting that PHM treatment may indirectly enhance SCFA-associated metabolic activity in the inflamed colon [71]. These compositional shifts suggest a functional transition toward a microbiota profile more favorable for SCFA production and epithelial barrier support.

A more detailed analysis at the species level demonstrated that PHM administration significantly reduced the relative abundance of *Bacteroides (B.) acidifaciens* (*p* ≤ 0.01, Fig. 6F), a species within the *Bacteroidaceae* family that has been associated with intestinal inflammation and IBD progression [73]. This bacterial species displays key characteristics that could classify it as a potential colitis-specific biomarker—or, at the very least, as a contributor to colitis development and progression. *B. acidifaciens* was recently shown to act as a mucin degrader *in vivo* [74]. Since mucin serves as a natural barrier in the healthy intestinal environment, its degradation can compromise epithelial integrity, promote microbial dysbiosis, and trigger inflammatory responses, suggesting that *B. acidifaciens* may directly exacerbate disease pathology. From a metabolic perspective, excessive mucin degradation can shift microbial energy utilization toward host-derived glycans rather than dietary polysaccharides, a process often associated with epithelial barrier erosion and sustained inflammation [75]. Thus, the reduction of *B. acidifaciens* abundance by PHM may contribute to preservation of the mucus layer and reinforcement of the intestinal barrier. In parallel, PHM treatment led to a marked increase in the abundance of PAC001120 (*p* ≤ 0.01), an unclassified species within the *Lachnospiraceae* family known for its capacity to ferment complex polysaccharides and produce beneficial SCFAs [71]. The enrichment of this taxon suggests a potential shift in microbial metabolic pathways toward enhanced polysaccharide fermentation and SCFA biosynthesis, which are known to support epithelial energy metabolism and anti-inflammatory signaling [71]. Interestingly, PAC001120 has also been reported to expand in murine models in association with systemic immune modulation via regulatory T cells and Th1 cell populations [76], suggesting that its enrichment may reflect or promote a more balanced mucosal immune environment. Taken together, although direct metagenomic or metabolomic analyses were not performed, the observed taxonomic shifts allow inference of functional metabolic changes, including increased SCFA-related fermentation pathways and reduced host-glycan degradation, which are closely linked to improved barrier integrity and immune homeostasis in the gut. These microbial shifts suggest that PHM contributes to rebalancing the gut microbiota by suppressing potentially pro-inflammatory species such as *B. acidifaciens*, while promoting beneficial taxa associated with SCFA production and immune regulation. Taken together, our findings support the potential of oral PHM to alleviate gut dysbiosis and help restore a healthier microbial composition under inflammatory conditions such as colitis.

## 4 Conclusion

In this study, we developed PHM incorporating an internal oil layer using a microfluidic-based triple emulsion strategy for the oral delivery of PTX in IBD. The precisely controlled multiphasic microfluidic fabrication enabled uniform production of PAA–PEGDA-shelled microcapsules with a protective oil barrier, which effectively shielded PTX from gastric acidity and supported its stability during gastrointestinal transit. This design allowed pH-triggered drug release under mildly alkaline colonic conditions, resulting in sustained gastrointestinal residence and preferential localization in the colon. Oral administration of PTX-loaded PHM alleviated DSS-induced colitis, as indicated by improvements in disease activity, histological features, inflammatory cell infiltration, and expression of pro-inflammatory mediators. In addition, PHM treatment was associated with alterations in gut microbial composition, including suppression of inflammation-associated taxa and enrichment of potentially beneficial bacterial populations. These findings suggest that PHM-mediated delivery enhances local PTX availability while limiting premature release in the upper gastrointestinal tract. Several limitations of the present study should be acknowledged. While changes in gut microbial composition were identified using 16S rRNA gene sequencing, direct metagenomic or metabolomic analyses were not performed, limiting mechanistic insight into functional microbial pathways and metabolite-level alterations associated with PHM treatment. Moreover, although short-term *in vivo* behavior and gastrointestinal distribution of PHM were evaluated, longer-term safety, degradation characteristics, and scalability under clinically relevant conditions remain to be fully established. Addressing these aspects in future studies will be important for advancing the translational potential of this platform. Overall, this work demonstrates the feasibility of a microfluidic-based PHM system for targeted oral delivery in IBD and provides a foundation for further optimization and evaluation toward clinical application.

## Data and materials availability

All data are available in the main text or the supplementary data.

## CRediT authorship contribution statement

**Ji-Yeon Park:** Data curation, Investigation, Methodology, Visualization, Writing – original draft. **Hye-Seon Jeong:** Data curation, Investigation, Methodology, Visualization, Writing – original draft. **Seong-Ryeong Lim:** Conceptualization, Data curation, Formal analysis, Methodology, Visualization. **Won-Kyo Jung:** Conceptualization, Data curation, Formal analysis, Methodology, Visualization. **Jae-Young Je:** Conceptualization, Data curation, Formal analysis, Methodology, Visualization. **Chang-Hyung Choi:** Conceptualization, Funding acquisition, Investigation, Supervision, Visualization, Writing – original draft, Writing – review & editing. **Sei-Jung Lee:** Conceptualization, Funding acquisition, Investigation, Supervision, Visualization, Writing – original draft, Writing – review & editing.

## Declaration of competing interest

The authors declare that they have no known competing financial interests or personal relationships that could have appeared to influence the work reported in this paper.

## Data Availability

Data will be made available on request.

## References

[1] Khor B., Gardet A., Xavier R.J. (2011). Genetics and pathogenesis of inflammatory bowel disease. Nature.

[2] Sturm A., Dignass A.U. (2008). Epithelial restitution and wound healing in inflammatory bowel disease. World J. Gastroenterol..

[3] Langhorst J., Wulfert H., Lauche R., Klose P., Cramer H., Dobos G.J., Korzenik J. (2015). Systematic review of complementary and alternative medicine treatments in inflammatory bowel diseases. J. Crohns Colitis.

[4] Weissman S., Sinh P., Mehta T.I., Thaker R.K., Derman A., Heiberger C., Qureshi N., Amrutiya V., Atoot A., Dave M., Tabibian J.H. (2020). Atherosclerotic cardiovascular disease in inflammatory bowel disease: the role of chronic inflammation. World J. Gastrointest. Pathophysiol..

[5] Kim E.J., Jeong H.S., Park J.Y., Je J.Y., Choi C.H., Lee S.J. (2025). The inflammatory bowel disease and gut microbiome are restored by employing metformin-loaded alginate-shelled microcapsules. J Control Release.

[6] Magri S., Paduano D., Chicco F., Cingolani A., Farris C., Delogu G., Tumbarello F., Lai M., Melis A., Casula L., Fantini M.C., Usai P. (2019). Nonalcoholic fatty liver disease in patients with inflammatory bowel disease: beyond the natural history. World J. Gastroenterol..

[7] Gunther C., Rothhammer V., Karow M., Neurath M., Winner B. (2021). The gut-brain axis in inflammatory bowel disease-current and future perspectives. Int. J. Mol. Sci..

[8] Atreya R., Neurath M.F. (2008). New therapeutic strategies for treatment of inflammatory bowel disease. Mucosal Immunol..

[9] Bahaa M.M., Hegazy S.K., Maher M.M., Bahgat M.M., El-Haggar S.M. (2024). Pentoxifylline in patients with ulcerative colitis treated with mesalamine by modulation of IL-6/STAT3, ZO-1, and S1P pathways: a randomized controlled double-blinded study. Inflammopharmacology.

[10] Lee H.J. (2022). Therapeutic potential of the combination of pentoxifylline and Vitamin-E in inflammatory bowel disease: inhibition of intestinal fibrosis. J. Clin. Med..

[11] Karatay E., Gul Utku O., Erdal H., Arhan M., Onal I.K., Ibis M., Ekinci O., Yilmaz Demirtas C., S G.D. (2017). Pentoxifylline attenuates mucosal damage in an experimental model of rat colitis by modulating tissue biomarkers of inflammation, oxidative stress, and fibrosis. Turk. J. Med. Sci..

[12] Chen Y.M., Tu C.J., Hung K.Y., Wu K.D., Tsai T.J., Hsieh B.S. (2003). Inhibition by pentoxifylline of TNF-alpha-stimulated fractalkine production in vascular smooth muscle cells: evidence for mediation by NF-kappa B down-regulation. Br. J. Pharmacol..

[13] Neuner P., Klosner G., Schauer E., Pourmojib M., Macheiner W., Grunwald C., Knobler R., Schwarz A., Luger T.A., Schwarz T. (1994). Pentoxifylline in vivo down-regulates the release of IL-1 beta, IL-6, IL-8 and tumour necrosis factor-alpha by human peripheral blood mononuclear cells. Immunology.

[14] Broderick C., Forster R., Abdel-Hadi M., Salhiyyah K. (2020). Pentoxifylline for intermittent claudication. Cochrane Database Syst. Rev..

[15] Aviado D.M., Porter J.M. (1984). Pentoxifylline: a new drug for the treatment of intermittent claudication. Mechanism of action, pharmacokinetics, clinical efficacy and adverse effects. Pharmacotherapy.

[16] Ward A., Clissold S.P. (1987). Pentoxifylline. A review of its pharmacodynamic and pharmacokinetic properties, and its therapeutic efficacy. Drugs.

[17] Sein Anand J., Wiergowski M., Wisniewski M.R., Kosmowska M., Kata M., Wozniak M.K. (2022). Fatal suicidal intoxication with pentoxifylline complicated by cardiovascular disorders. Toxics.

[18] Nugent S.G., Kumar D., Rampton D.S., Evans D.F. (2001). Intestinal luminal pH in inflammatory bowel disease: possible determinants and implications for therapy with aminosalicylates and other drugs. Gut.

[19] Xie X., Wang Y., Deng B., Blatchley M.R., Lan D., Xie Y., Lei M., Liu N., Xu F., Wei Z. (2024). Matrix metalloproteinase-responsive hydrogels with tunable retention for on-demand therapy of inflammatory bowel disease. Acta Biomater..

[20] Liu Y., Huang J., Li S., Li Z., Chen C., Qu G., Chen K., Teng Y., Ma R., Wu X., Ren J. (2024). Advancements in hydrogel-based drug delivery systems for the treatment of inflammatory bowel disease: a review. Biomater. Sci..

[21] Xie X., Chen X., Zhou J., Wang T., Yang G., Han F., Wei Z. (2025). Dynamic hydrogels with tunable mechanics for 3D organoid derivation. Small.

[22] Liu L., Yao W., Rao Y., Lu X., Gao J. (2017). pH-Responsive carriers for oral drug delivery: challenges and opportunities of current platforms. Drug Deliv..

[23] Weiss M., Frohnmayer J.P., Benk L.T., Haller B., Janiesch J.W., Heitkamp T., Borsch M., Lira R.B., Dimova R., Lipowsky R., Bodenschatz E., Baret J.C., Vidakovic-Koch T., Sundmacher K., Platzman I., Spatz J.P. (2018). Sequential bottom-up assembly of mechanically stabilized synthetic cells by microfluidics. Nat. Mater..

[24] Shum H.C., Kim J.W., Weitz D.A. (2008). Microfluidic fabrication of monodisperse biocompatible and biodegradable polymersomes with controlled permeability. J. Am. Chem. Soc..

[25] Kim S.H., Kim J.W., Kim D.H., Han S.H., Weitz D.A. (2013). Polymersomes containing a hydrogel network for high stability and controlled release. Small.

[26] Amstad E., Kim S.H., Weitz D.A. (2012). Photo- and thermoresponsive polymersomes for triggered release. Angew Chem. Int. Ed. Engl..

[27] Tan H., Guo S., Dinh N.D., Luo R., Jin L., Chen C.H. (2017). Heterogeneous multi-compartmental hydrogel particles as synthetic cells for incompatible tandem reactions. Nat. Commun..

[28] Seiffert S., Thiele J., Abate A.R., Weitz D.A. (2010). Smart microgel capsules from macromolecular precursors. J. Am. Chem. Soc..

[29] Aibani N., Khan T.N., Callan B. (2020). Liposome mimicking polymersomes; A comparative study of the merits of polymersomes in terms of formulation and stability. Int J Pharm X.

[30] Li W., Zhang L., Ge X., Xu B., Zhang W., Qu L., Choi C.H., Xu J., Zhang A., Lee H., Weitz D.A. (2018). Microfluidic fabrication of microparticles for biomedical applications. Chem. Soc. Rev..

[31] Lee T.Y., Choi T.M., Shim T.S., Frijns R.A., Kim S.H. (2016). Microfluidic production of multiple emulsions and functional microcapsules. Lab Chip.

[32] Abbaspourrad A., Carroll N.J., Kim S.H., Weitz D.A. (2013). Polymer microcapsules with programmable active release. J. Am. Chem. Soc..

[33] Windbergs M., Zhao Y., Heyman J., Weitz D.A. (2013). Biodegradable core-shell carriers for simultaneous encapsulation of synergistic actives. J. Am. Chem. Soc..

[34] Choi C.H., Weitz D.A., Lee C.S. (2013). One step formation of controllable complex emulsions: from functional particles to simultaneous encapsulation of hydrophilic and hydrophobic agents into desired position. Adv Mater.

[35] Xu W., Ledin P.A., Iatridi Z., Tsitsilianis C., Tsukruk V.V. (2016). Multicompartmental microcapsules with orthogonal programmable two-way sequencing of hydrophobic and hydrophilic cargo release. Angew Chem. Int. Ed. Engl..

[36] Dinh N.D., Kukumberg M., Nguyen A.T., Keramati H., Guo S., Phan D.T., Ja'Afar N.B., Birgersson E., Leo H.L., Huang R.Y., Kofidis T., Rufaihah A.J., Chen C.H. (2020). Functional reservoir microcapsules generated via microfluidic fabrication for long-term cardiovascular therapeutics. Lab Chip.

[37] Xiao Z., Wei T., Ge R., Li Q., Liu B., Ji Z., Chen L., Zhu J., Shen J., Liu Z., Huang Y., Yang Y., Chen Q. (2022). Microfluidic production of zwitterion coating microcapsules with low foreign body reactions for improved islet transplantation. Small.

[38] Arriaga L.R., Datta S.S., Kim S.H., Amstad E., Kodger T.E., Monroy F., Weitz D.A. (2014). Ultrathin shell double emulsion templated giant unilamellar lipid vesicles with controlled microdomain formation. Small.

[39] Kim S.H., Kim J.W., Cho J.C., Weitz D.A. (2011). Double-emulsion drops with ultra-thin shells for capsule templates. Lab Chip.

[40] Jeong H.S., Kim E., Nam C., Choi Y., Lee Y.J., Weitz D.A., Lee H., Choi C.H. (2021). Hydrogel microcapsules with a thin oil layer: smart triggered release via diverse stimuli. Adv. Funct. Mater..

[41] Kim D.W., Jeong H.S., Kim E., Lee H., Choi C.H., Lee S.J. (2022). Oral delivery of stem-cell-loaded hydrogel microcapsules restores gut inflammation and microbiota. J Control Release.

[42] Mukamel E., Servadio C. (1989). [importance and accuracy of the diagnosis of cancer confined to the prostate]. Harefuah.

[43] Lee H., Choi C.H., Abbaspourrad A., Wesner C., Caggioni M., Zhu T., Nawar S., Weitz D.A. (2016). Fluorocarbon oil reinforced triple emulsion drops. Adv Mater.

[44] Wirtz S., Neufert C., Weigmann B., Neurath M.F. (2007). Chemically induced mouse models of intestinal inflammation. Nat. Protoc..

[45] Cooper H.S., Murthy S., Shah R., Sedergran D.J.L.i., methods a.j.o.t., pathology (1993). Clinicopathologic study of dextran sulfate sodium experimental murine colitis.

[46] Nava P., Koch S., Laukoetter M.G., Lee W.Y., Kolegraff K., Capaldo C.T., Beeman N., Addis C., Gerner-Smidt K., Neumaier I.J.I. (2010). Interferon-γ regulates intestinal epithelial homeostasis through converging β-catenin signaling pathways.

[47] Hu Y., Wang Y., Guan R., Zhang C., Shao X., Yue Q. (2021). Construction of a ratio fluorescence assay of 5-aminosalicylic acid based on its aggregation induced emission with blue emitting N/P-codoped carbon dots. RSC Adv..

[48] Fadrosh D.W., Ma B., Gajer P., Sengamalay N., Ott S., Brotman R.M., Ravel J. (2014). An improved dual-indexing approach for multiplexed 16S rRNA gene sequencing on the Illumina MiSeq platform. Microbiome.

[49] Yoon S.H., Ha S.M., Kwon S., Lim J., Kim Y., Seo H., Chun J. (2017). Introducing EzBioCloud: a taxonomically united database of 16S rRNA gene sequences and whole-genome assemblies. Int. J. Syst. Evol. Microbiol..

[50] Fu L., Niu B., Zhu Z., Wu S., Li W. (2012). CD-HIT: accelerated for clustering the next-generation sequencing data. Bioinformatics.

[51] Edgar R.C.J.B. (2010). Search and clustering orders of magnitude faster than BLAST.

[52] Swift T., Swanson L., Geoghegan M., Rimmer S. (2016). The pH-responsive behaviour of poly(acrylic acid) in aqueous solution is dependent on molar mass. Soft Matter.

[53] Abell T.L., Camilleri M., Donohoe K., Hasler W.L., Lin H.C., Maurer A.H., McCallum R.W., Nowak T., Nusynowitz M.L., Parkman H.P., Shreve P., Szarka L.A., Snape W.J., Ziessman H.A., American N., Motility S., M. the Society of Nuclear (2008). Consensus recommendations for gastric emptying scintigraphy: a joint report of the American Neurogastroenterology and Motility Society and the Society of nuclear Medicine. J. Nucl. Med. Technol..

[54] Castro-Dopico T., Dennison T.W., Ferdinand J.R., Mathews R.J., Fleming A., Clift D., Stewart B.J., Jing C., Strongili K., Labzin L.I., Monk E.J.M., Saeb-Parsy K., Bryant C.E., Clare S., Parkes M., Clatworthy M.R. (2019). Anti-commensal IgG drives intestinal inflammation and type 17 immunity in ulcerative colitis. Immunity.

[55] Sun D., Bai R., Zhou W., Yao Z., Liu Y., Tang S., Ge X., Luo L., Luo C., Hu G.F., Sheng J., Xu Z. (2021). Angiogenin maintains gut microbe homeostasis by balancing alpha-Proteobacteria and Lachnospiraceae. Gut.

[56] Franzosa E.A., Sirota-Madi A., Avila-Pacheco J., Fornelos N., Haiser H.J., Reinker S., Vatanen T., Hall A.B., Mallick H., McIver L.J., Sauk J.S., Wilson R.G., Stevens B.W., Scott J.M., Pierce K., Deik A.A., Bullock K., Imhann F., Porter J.A., Zhernakova A., Fu J., Weersma R.K., Wijmenga C., Clish C.B., Vlamakis H., Huttenhower C., Xavier R.J. (2019). Gut microbiome structure and metabolic activity in inflammatory bowel disease. Nat. Microbiol..

[57] Kruger A., Matulla B., Wolzt M., Pieh S., Strenn K., Findl O., Eichler H.G., Schmetterer L. (1998). Short-term oral pentoxifylline use increases choroidal blood flow in patients with age-related macular degeneration. Arch. Ophthalmol..

[58] Reagan-Shaw S., Nihal M., Ahmad N. (2008). Dose translation from animal to human studies revisited. FASEB J..

[59] Liu H., Cai Z., Wang F., Hong L., Deng L., Zhong J., Wang Z., Cui W.J.A.S. (2021). Colon‐Targeted adhesive Hydrogel microsphere for regulation of gut immunity and flora. Adv. Sci..

[60] Liu T., Zhang L., Joo D., Sun S.C. (2017). NF-kappaB signaling in inflammation. Signal Transduct. Targeted Ther..

[61] Schreiber S., Nikolaus S., Hampe J. (1998). Activation of nuclear factor kappa B inflammatory bowel disease. Gut.

[62] Rogler G., Brand K., Vogl D., Page S., Hofmeister R., Andus T., Knuechel R., Baeuerle P.A., Scholmerich J., Gross V. (1998). Nuclear factor kappaB is activated in macrophages and epithelial cells of inflamed intestinal mucosa. Gastroenterology.

[63] Wang D., Dubois R.N. (2010). The role of COX-2 in intestinal inflammation and colorectal cancer. Oncogene.

[64] Mu K., Yu S., Kitts D.D. (2019). The role of nitric oxide in regulating intestinal redox status and intestinal epithelial cell functionality. Int. J. Mol. Sci..

[65] Li M.O., Wan Y.Y., Flavell R.A. (2007). T cell-produced transforming growth factor-beta1 controls T cell tolerance and regulates Th1- and Th17-cell differentiation. Immunity.

[66] Burgueno J.F., Abreu M.T. (2020). Epithelial Toll-like receptors and their role in gut homeostasis and disease. Nat. Rev. Gastroenterol. Hepatol..

[67] Lu Y., Li X., Liu S., Zhang Y., Zhang D. (2018). Toll-like receptors and inflammatory bowel disease. Front. Immunol..

[68] Caruso R., Lo B.C., Nunez G. (2020). Host-microbiota interactions in inflammatory bowel disease. Nat. Rev. Immunol..

[69] Hou K., Wu Z.X., Chen X.Y., Wang J.Q., Zhang D., Xiao C., Zhu D., Koya J.B., Wei L., Li J., Chen Z.S. (2022). Microbiota in health and diseases. Signal Transduct. Targeted Ther..

[70] Stojanov S., Berlec A., Strukelj B. (2020). The influence of probiotics on the Firmicutes/Bacteroidetes ratio in the treatment of obesity and inflammatory bowel disease. Microorganisms.

[71] Vacca M., Celano G., Calabrese F.M., Portincasa P., Gobbetti M., De Angelis M. (2020). The controversial role of human gut Lachnospiraceae. Microorganisms.

[72] Huang Y.L., Chassard C., Hausmann M., von Itzstein M., Hennet T. (2015). Sialic acid catabolism drives intestinal inflammation and microbial dysbiosis in mice. Nat. Commun..

[73] Busbee P.B., Menzel L., Alrafas H.R., Dopkins N., Becker W., Miranda K., Tang C., Chatterjee S., Singh U., Nagarkatti M., Nagarkatti P.S. (2020). Indole-3-carbinol prevents colitis and associated microbial dysbiosis in an IL-22-dependent manner. JCI Insight.

[74] Berry D., Stecher B., Schintlmeister A., Reichert J., Brugiroux S., Wild B., Wanek W., Richter A., Rauch I., Decker T., Loy A., Wagner M. (2013). Host-compound foraging by intestinal microbiota revealed by single-cell stable isotope probing. Proc. Natl. Acad. Sci. U. S. A..

[75] Yamaguchi M., Yamamoto K. (2023). Mucin glycans and their degradation by gut microbiota. Glycoconj. J..

[76] Han S.K., Shin Y.J., Lee D.Y., Kim K.M., Yang S.J., Kim D.S., Choi J.W., Lee S., Kim D.H. (2021). Lactobacillus rhamnosus HDB1258 modulates gut microbiota-mediated immune response in mice with or without lipopolysaccharide-induced systemic inflammation. BMC Microbiol..

