# Supplementary material for: Triple-emulsion microfluidic Core–Shell hydrogel microcapsules for oral pentoxifylline Delivery: Ameliorating colitis and rebalancing gut microbiome
Source: Mater Today Bio. 2026 Feb 2;37:102881. doi: 10.1016/j.mtbio.2026.102881 (PMC12890840; doi:10.1016/j.mtbio.2026.102881)
Supplement: Multimedia component 1 [file mmc1.docx]

Supplementary data

**Triple‑Emulsion Microfluidic Core–Shell Hydrogel Microcapsules for Oral Pentoxifylline Delivery: Ameliorating Colitis and Rebalancing Gut Microbiome**

Ji-Yeon Park ^a,1^, Hye-Seon Jeong ^b,1^, Seong-Ryeong Lim ^a^, Won-Kyo Jung ^c^,

Jae-Young Je ^a^, Chang-Hyung Choi ^b,*^, Sei-Jung Lee ^a,*^

^a^ *Major of Human Bio-convergence, Division of Smart Healthcare, Pukyong National University, Busan, 48513, Republic of Korea*

^b^ *School of Chemical Engineering, Yeungnam University, 280 Daehak-ro, Gyeongsan, Gyeongbuk 38541, Republic of Korea*

^c^ *Major of Biomedical Engineering, Division of Smart Healthcare, Pukyong National University, Busan 48513, Republic of Korea*

*** Corresponding authors**: Sei-Jung Lee Ph.D. and Chang-Hyung Choi, Ph.D.

Major of Human Bio-convergence, Pukyong National University, 45 Yongso-ro, Information Technology and Convergence Building, Room 414, Busan 48513, Republic of Korea

E-mail: seijung1@pknu.ac.kr; Tel: 82-51-629-4636; Fax: 82-51-629-4639

^1^ These authors contributed equally to this work.

1. Supplementary tables

**Table S1. Scoring of disease activity index (DAI).**

| **Score** | **Weight loss (%)** | **Stool consistency** | **Occult/gross bleeding** |
| --- | --- | --- | --- |
| 0 | None | Normal stools ^a)^ | Negative |
| 1 | 1 – 5 | Loose stools ^b)^ | Negative |
| 2 | 5 – 10 | Loose stools | Hemoccult positive |
| 3 | 10 – 20 | Diarrhea ^c)^ | Hemoccult positive |
| 4 | > 20 | Diarrhea | Gross bleeding |

^a)^Normal stools : well formed pellets; ^b)^Loose stools : pasty and semi-formed stools which do not stick to the anus; ^c)^Diarrhea : liquid stools that stick to the anus. The DAI value is combined the scores of weight loss, stool consistency, and bleeding [45].

**Table S2.** PCR primer sequences

| **Gene** | **Identification** | **Primer sequence, 5`-3`** | **Size (bp)** |
| --- | --- | --- | --- |
| IL-1β | Forward | GGGCTGCTTCCAAACCTTTG | 287 |
|  | Reverse | GGAGCCTGTAGTGCAGTTGT |  |
| IL-6 | Forward | ACCACTTCACAAGTCGGAGG | 296 |
|  | Reverse | TCTCTCTGAAGGACTCTGGCT |  |
| TNF-α | Forward | CACAGAAAGCATGATCCGCG | 211 |
|  | Reverse | ACTGATGAGAGGGAGGCCAT |  |
| TLR-4 | Forward | GCTTTCACCTCTGCCTTCAC | 259 |
|  | Reverse | AGGCGATACAATTCCACCTG |  |
| TLR-5 | Forward | CTTTTCAAGGTCTGCCCCAT | 256 |
|  | Reverse | GACAGTGTTTTGCCCTGCAG |  |
| TLR-9 | Forward | TCGCTTTGTGGATTGTCAG | 266 |
|  | Reverse | GGCTCAGGCTAAGACACTGG |  |
| β-actin | Forward | AGCCATGTACGTAGCCATCC | 228 |
|  | Reverse | CTCTCAGCTGTGGTGGTGAA |  |

**Table S3.** PCR primer sequences

| **Gene** | **Identification** | **Primer sequence, 5`-3`** | **Size (bp)** |
| --- | --- | --- | --- |
| Regions V3-V4 of 16S rRNA | Forward | TCGTCGGCAGCGTCAGATGTGTATAAGAGACAGCCTACGGGNGGCWGCAG | 50 |
|  | Reverse | GTCTCGTGGGCTCGGAGATGTGTATAAGAGACAGGACTACHVGGGTATCTAATCC |  |

2. Supplementary figures


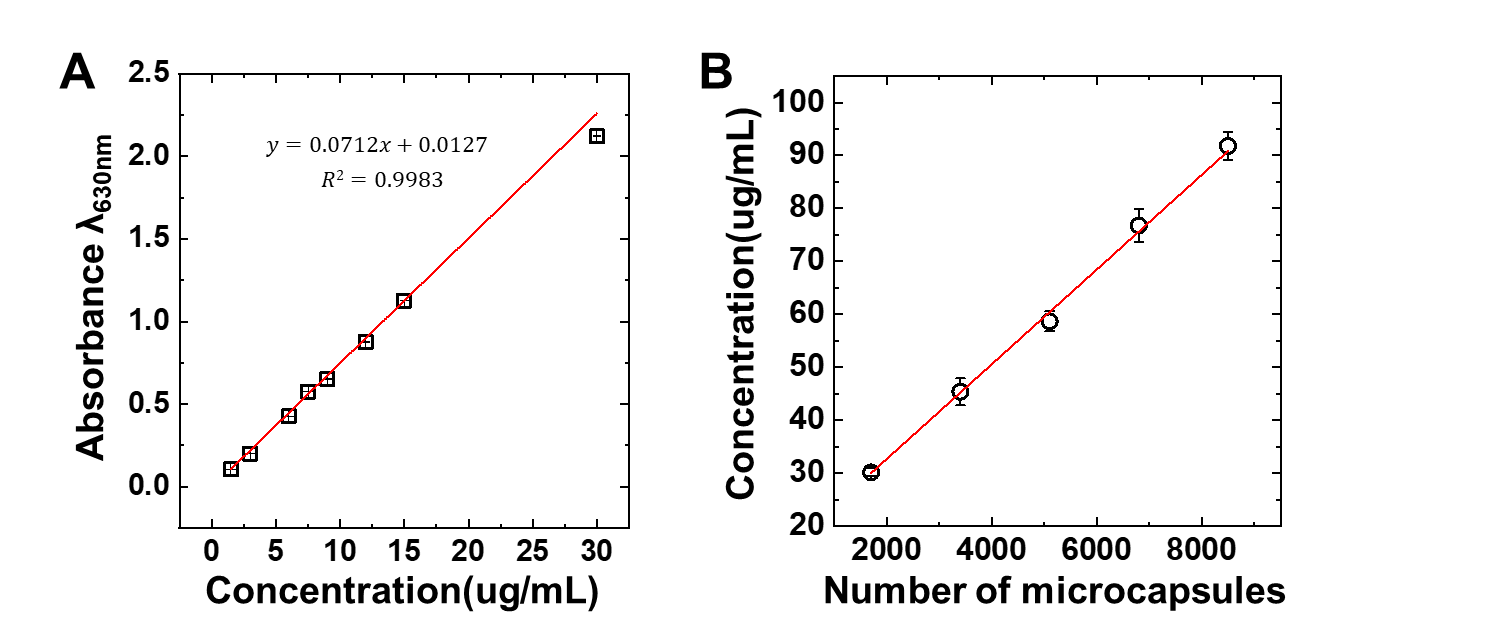


**Figure S1. Quantitative analysis of blue dye loading and release.** (A) Calibration curve of erioglaucine disodium salt (blue dye) showing the linear relationship between absorbance at 630 nm and dye concentration. (B) Linear correlation between the number of hydrogel microcapsules and the concentration of blue dye released into a fixed volume of buffer, demonstrating quantitative and reproducible control of the released amount based on capsule number. Data represent mean ± SD (n = 3).


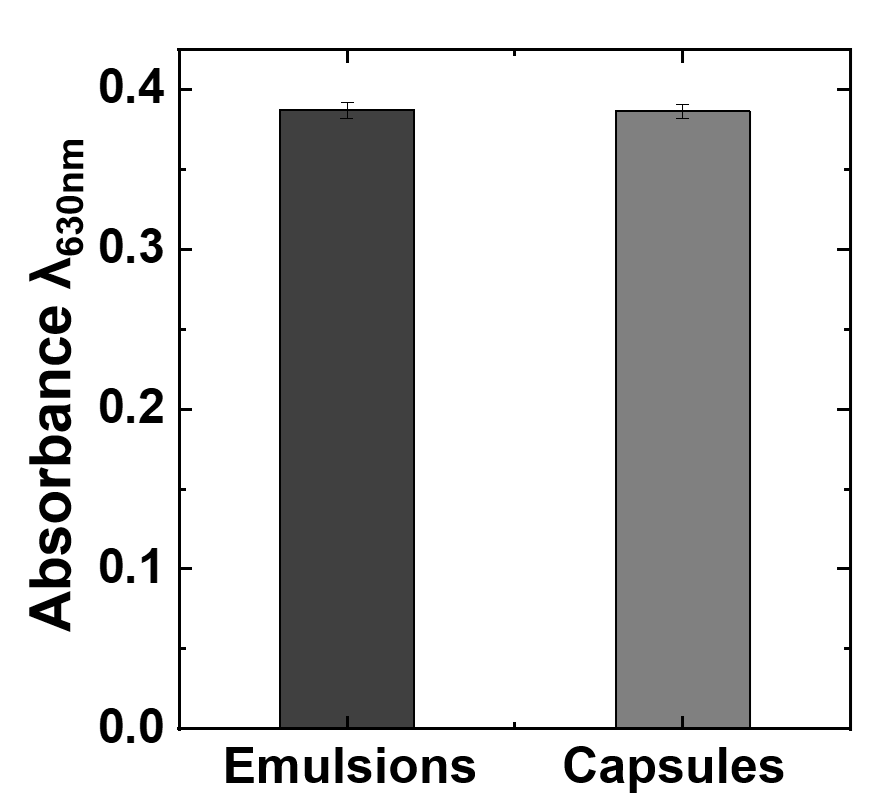


**Figure S2. Evaluation of encapsulation efficiency.** Absorbance comparison between the dye-containing emulsion prior to encapsulation and an equivalent number of hydrogel microcapsules after complete dye release, indicating an encapsulation efficiency of approximately 99.6%. Data are shown as mean ± SD (n = 3).


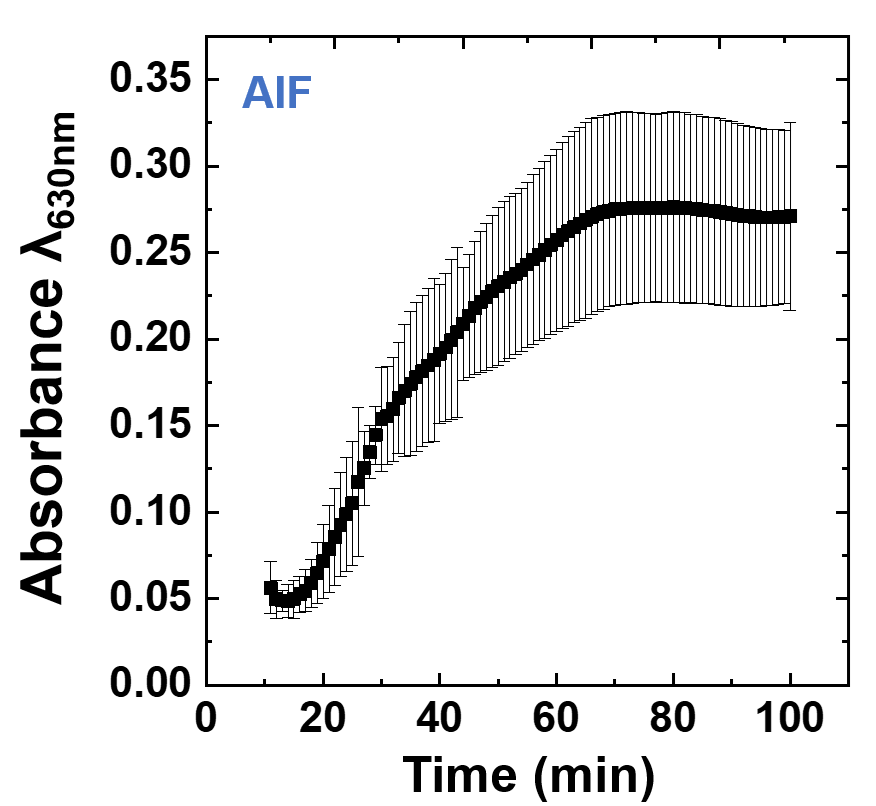


**Figure S3. *In vitro* release profiles.** Time-dependent release profile of the encapsulated model compound under artificial intestinal fluid (AIF, pH 7.5) conditions.

**
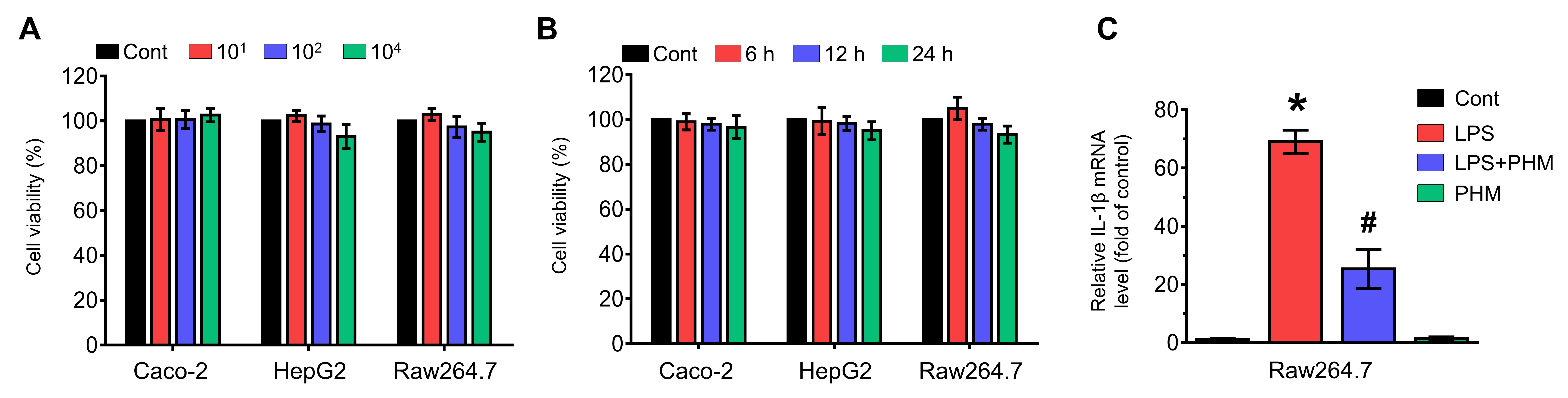
**

**Figure S4. Biocompatibility of PHM.** (A) Human gastrointestinal epithelial Caco-2 cells, human epithelial hepatocellular HepG2 cells, and murine macrophage RAW 264.7 cells were treated with increasing concentrations of PHM (10^1^ ~ 10^4^ capsules/mL) for 24 h, and cell viability was assessed using the EZ-CYTOX assay (n = 3). (B) Time-dependent effects of PHM (1 × 10^4^ capsules/mL) on the viability of the indicated cell types were evaluated at different time points (n = 3). (C) RAW 264.7 cells were stimulated with lipopolysaccharide (LPS, 1 μg/mL) in the presence or absence of PHM (10^4^ capsules/mL) for 24 h, and IL-1β mRNA expression levels were analyzed. **p* ≤ 0.001 vs. control. #*p* ≤ 0.01 vs. LPS alone. n = 3.

**
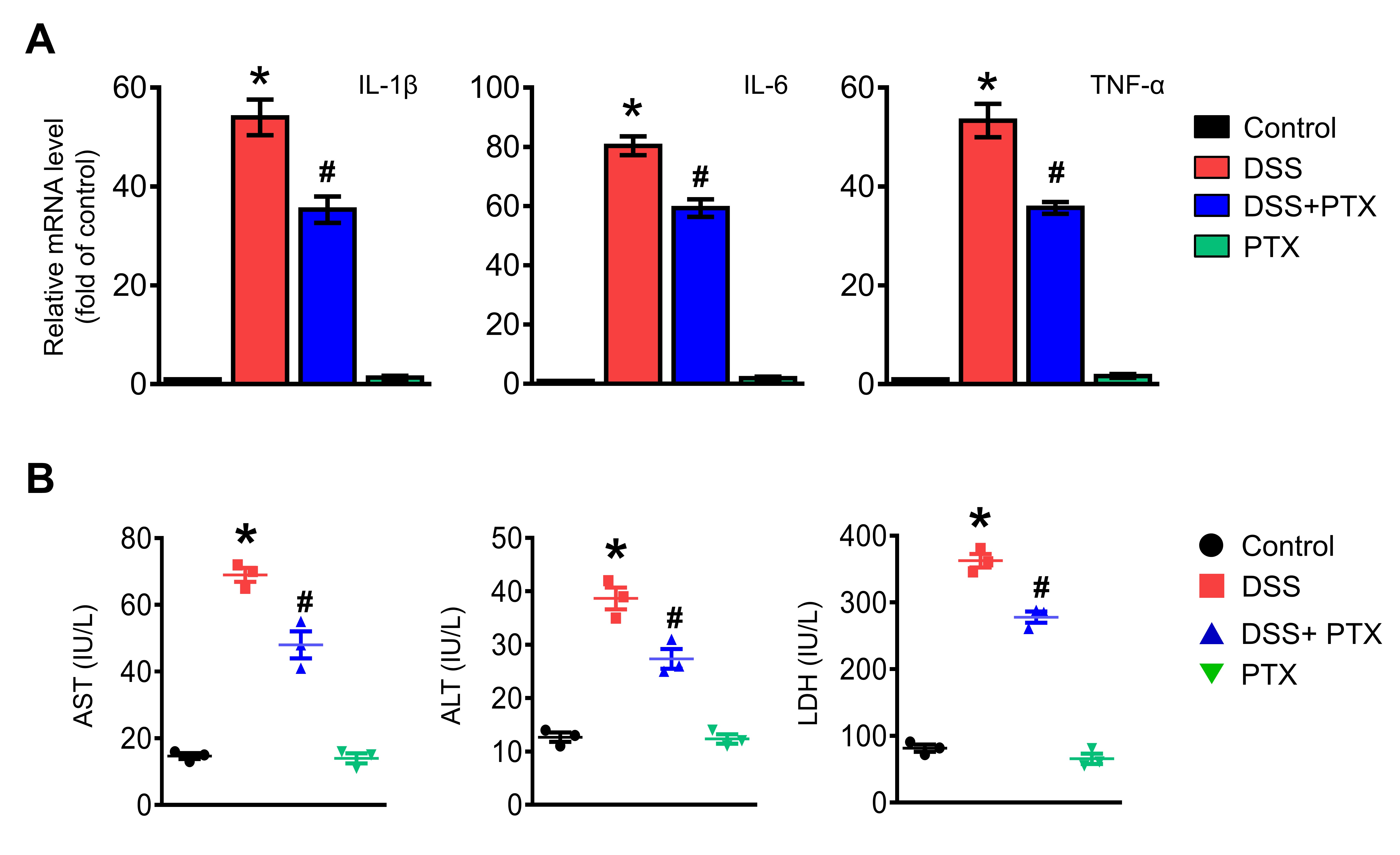
**

**Figure S5. Effects of PTX in a DSS-induced colitis model.** (A) Male ICR mice (n = 5) were orally administered PTX (100 mg/kg) every other day for 14 days. From day 7, 4% DSS was added to the drinking water for 7 days to induce colitis, while control mice received regular drinking water without DSS. The levels of pro-inflammatory cytokines (IL-1β, IL-6, and TNF-α) were measured to determine the modulatory effects of PTX. **p* ≤ 0.001 vs Control, #*p* ≤ 0.05 vs DSS. (B) Serum levels of tissue injury markers (AST, ALT, and LDH) are shown to evaluate systemic toxicity. **p* ≤ 0.001 vs Control, #*p* ≤ 0.01 vs DSS. n = 3.

**
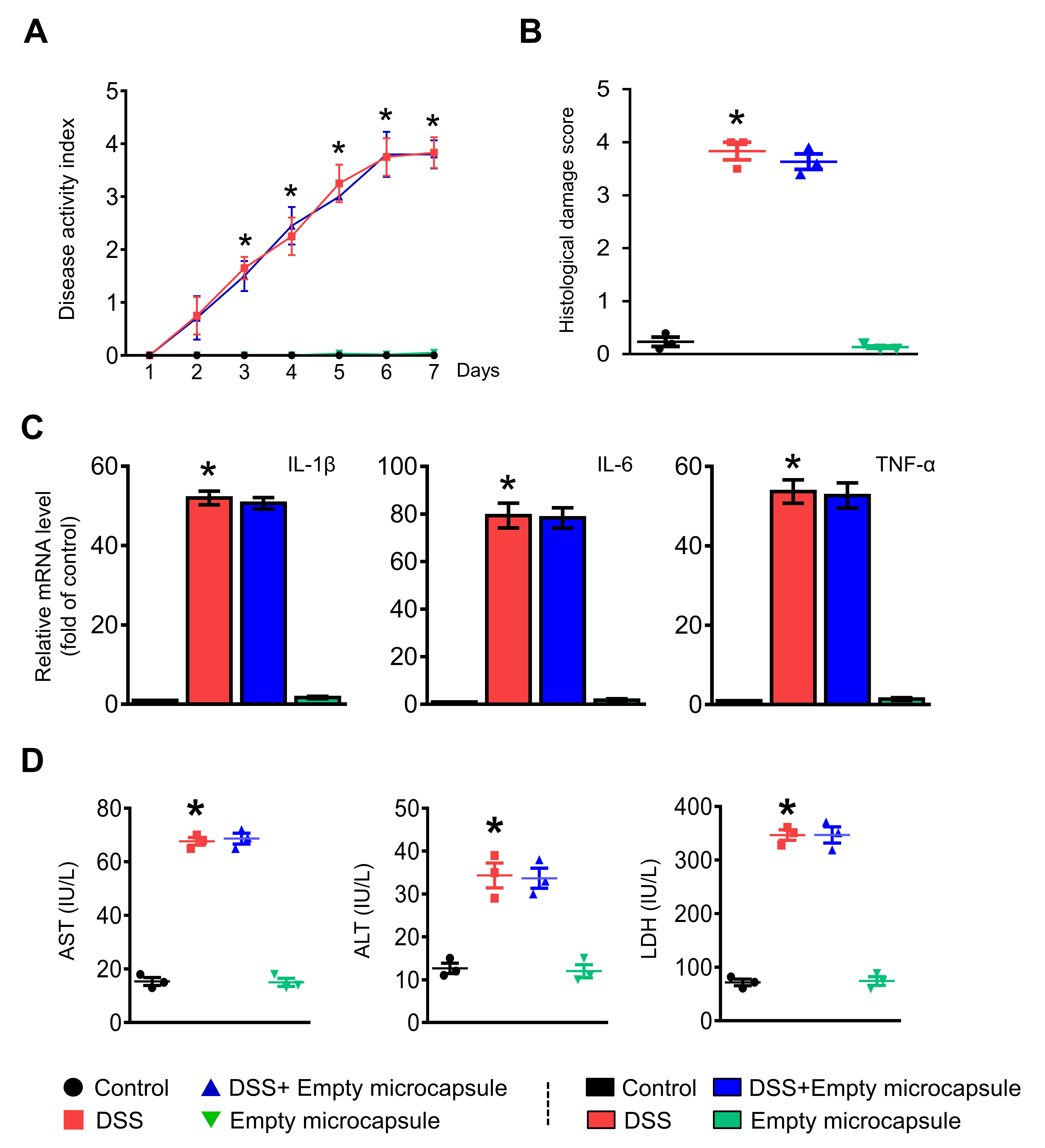
**

**Figure S6. Effects of empty microcapsules in a DSS-induced colitis model.** (A) Male ICR mice (n = 5) were orally administered empty microcapsules (1 × 10⁴ capsules/mL) every other day for 14 days. From day 7, 4% DSS was added to the drinking water for 7 days to induce colitis, while control mice received regular drinking water without DSS. DAI scores were recorded throughout the experimental period. **p* ≤ 0.01 vs. Control. (B) Histological damage scores of colonic tissues were evaluated. **p* ≤ 0.01 vs. Control. n = 3. (C) Levels of pro-inflammatory cytokines (IL-1β, IL-6, and TNF-α) were measured to assess inflammatory responses following empty microcapsule administration. **p* ≤ 0.001 vs. Control. n = 5. (D) Serum levels of tissue injury markers (AST, ALT, and LDH) were measured to evaluate systemic toxicity. **p* ≤ 0.001 vs. Control. n = 3.
